# Vitrification-Controlled Curing of Epoxidized Linseed Oil Resins: Kinetics, TTT Analysis, and Thermomechanical Performance with Anhydride and Amine Curing Agents

**DOI:** 10.1021/acsomega.6c05665

**Published:** 2026-09-21

**Authors:** Cristiane da Silva Fonseca, Maurício Carpe Diem Ferreira Xavier, Otávio Bianchi, Sandro Campos Amico, Cesar Liberato Petzhold

**Affiliations:** † Postgraduate Program in Mining, Metallurgical and Materials Engineering, Federal University of Rio Grande do Sul, Avenida Bento Gonçalves 9500, Porto Alegre 91501-970, Brazil; ‡ Postgraduate Program in Chemistry, Federal University of Rio Grande do Sul, Avenida Bento Gonçalves 9500, Porto Alegre 91501-970, Brazil

## Abstract

Epoxidized linseed oil (ELO) is a promising biobased alternative to petrochemical epoxy precursors, with a curing behavior strongly influenced by the chemistry of the curing agent and the resulting network architecture. In this study, ELO was synthesized via the Shi epoxidation approach and subsequently cured with either methyltetrahydrophthalic anhydride (ELO-AD) or a fatty amine agent (ELO-AM). Although epoxy–amine reactions exhibit lower activation energies (19–26 kJ·mol^–1^) than the epoxy–anhydride system (40–45 kJ·mol^–1^), the overall curing efficiency is governed by vitrification and diffusion constraints rather than intrinsic chemical reactivity. The anhydride-cured system forms a dense network with higher glass transition temperature (*T*_g_ = 46–94 °C), higher cross-link density (υ = 1.19 × 10^3^ mol·m^–3^), and gel content (97.95 ± 0.16%) together with rapid viscosity growth (*k*_η_ up to 0.83 min^–1^ at 140 °C), resulting in shorter characteristic times in time–temperature–transformation (TTT) diagrams. The amine-cured system forms a more flexible, lower *T*_g_ (−10 to 19 °C) network with lower υ (4.77 × 10^2^ mol·m^–3^), and gel content (79.87 ± 4.45%), consistent with a less interconnected netword and greater diffusion limitations at advanced conversion. These structural and kinetic differences translate directly into macroscopic performance. ELO-AD exhibits superior Young’s modulus and strength (2.0 GPa and ≈ 46 MPa, respectively), whereas ELO-AM shows enhanced ductility (strain at break ≈ 26%) and higher thermal stability (DTG peak ≈ 485 °C). Overall, this study establishes quantitative structure–process–property relationships for ELO-based epoxy systems, with practical guidelines for selecting curing agents and processing windows for sustainable thermoset resins.

## Graphical abstract

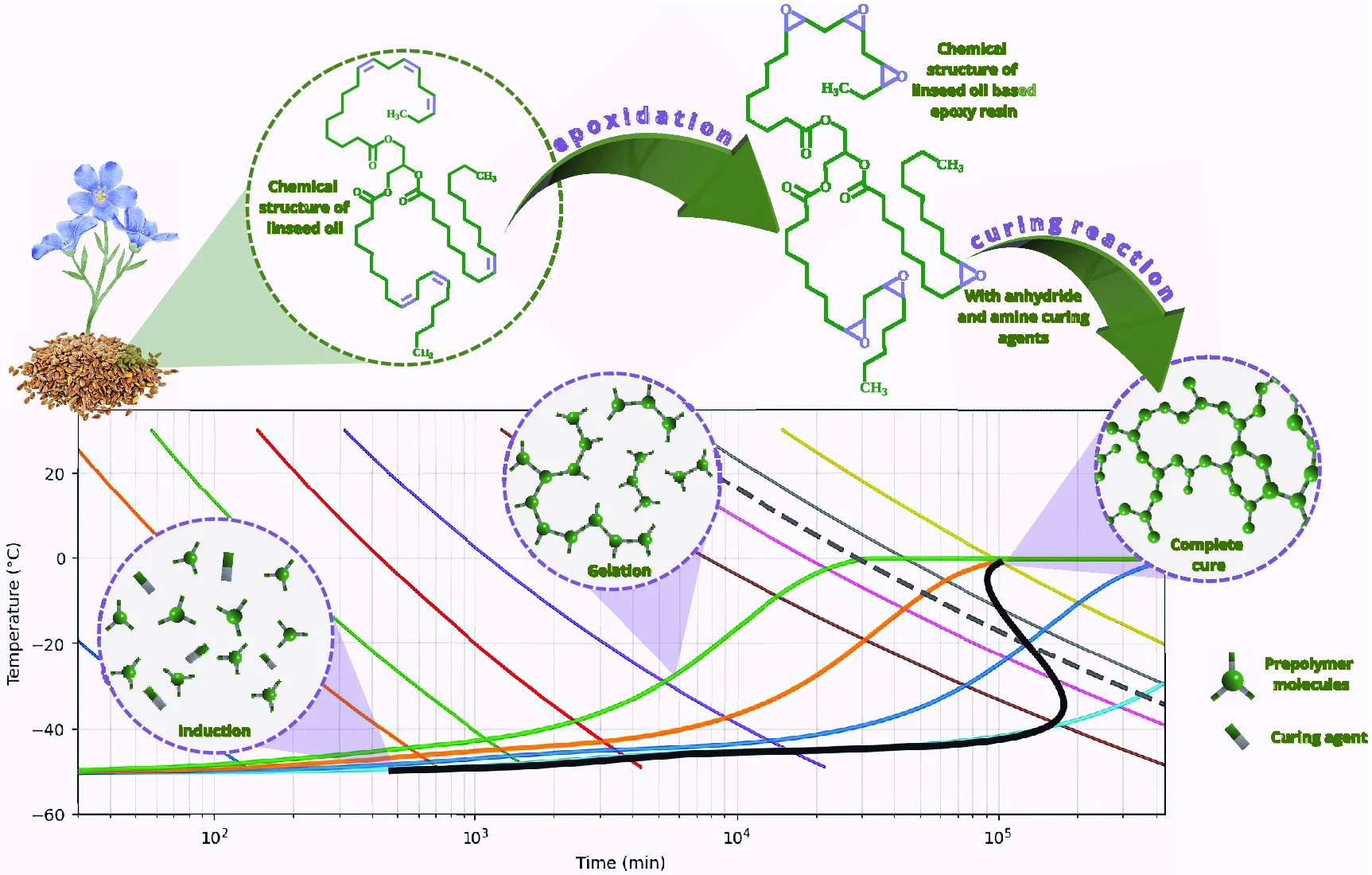

## Introduction

Epoxy resins constitute a cornerstone of modern thermosetting materials, owing to their ability to form densely cross-linked networks with high mechanical strength, thermal stability, and chemical resistance. These attributes originate from the high reactivity of epoxide groups combined with the structural rigidity typically imparted by aromatic backbones and multifunctional curing agents. Consequently, epoxy systems have been extensively employed in high-performance applications, including fiber-reinforced composites. Nevertheless, the overwhelming dominance of petrochemical-based formulations, particularly those derived from diglycidyl ether of bisphenol A (DGEBA), has raised significant environmental and health concerns, including toxicity, endocrine disruption, and environmental persistence.^1,2^

Biobased epoxy resins have emerged as alternative materials to reduce dependence on fossil resources and expand the design space of epoxy networks beyond the constraints imposed by rigid aromatic structures. Renewable feedstocks such as lignin, tannins, cardanol, terpenes, furans, and vegetable oils have been investigated as epoxy precursors, each with particular chemical architectures and reactivity patterns.^3–7^

Vegetable oils are particularly attractive due to their high availability, low toxicity, biodegradability, and intrinsic multifunctionality. They are molecularly characterized by a triglyceride composed of long-chain fatty acids that are predominantly aliphatic and often highly unsaturated. Epoxidation of carbon–carbon double bonds converts these intrinsically flexible molecules into multifunctional epoxy precursors. In contrast to conventional aromatic epoxy monomers, epoxidized vegetable oils combine pronounced segmental mobility with sterically constrained epoxy functionalities randomly distributed along extended aliphatic chains This structural duality governs curing kinetics and network development, often resulting in lower glass transition temperature (*T*_g_), broader curing exotherm, and increased susceptibility to diffusion-controlled regimes during cross-linking.^8,9^

Within this class of materials, linseed oil (LO) extracted from *Linum usitatissimum* stands out as an especially attractive epoxy precursor due to its high α-linolenic acid content, which provides a high degree of unsaturation and enables efficient epoxidation, yielding high epoxy functionality per molecule. The spatial distribution of double bonds along the fatty acid chains reduces steric hindrance during chemical modification, favoring the selective formation of oxirane rings.^10,11^ While these features make epoxidized linseed oil (ELO) a versatile platform for the development of biobased epoxy networks, they also introduce molecular heterogeneity and higher backbone flexibility compared to conventional DGEBA systems.

The curing of ELO-based resins is more complex and cannot be described by a single, uniform reaction pathway. The combination of a flexible aliphatic backbone and a heterogeneous distribution of epoxy groups leads to multistep curing mechanisms, in which chemical reaction kinetics, segmental mobility, and diffusion processes are strongly coupled. Under these conditions, the nature of the curing agent becomes decisive in governing network formation. Anhydrides, acids, and amines differ not only in their intrinsic reactivity toward epoxy groups but also in the stiffness, functionality, and secondary interactions they introduce into the evolving network, thereby modulating cross-link density, vitrification, and final thermomechanical properties.^12,13^

In epoxy–anhydride systems initiated by tertiary amines, curing typically proceeds via an anionic ring-opening mechanism, involving the formation of alkoxide and carboxylate intermediates that propagate via competing esterification and etherification reactions. These pathways are generally autocatalytic and exhibit strong temperature dependence, leading to a rapid acceleration of network formation once a critical concentration of reactive species and sufficient thermal energy are reached.^9,14^ In contrast, epoxy–amine systems are governed by the nucleophilic attack of amine to epoxide rings, yielding hydroxyl groups and secondary or tertiary amines. In biobased epoxy systems from vegetable oils, this is frequently accompanied by aminolysis reactions, in which amines react with ester groups within the triglyceride backbone, generating amide linkages and altering both the effective cross-link density and the topology of the developing network.^8,13^

The coexistence of these distinct and sometimes competing reaction pathways poses a challenge in the design of sustainable epoxy thermosets. High cross-link densities are associated with enhanced thermal stability and mechanical strength, but excessive rigidity can restrict molecular mobility, narrow processing windows, and reduce toughness. Conversely, curing agents that yield greater chain flexibility enhance deformability and energy dissipation, but often at the expense of stiffness and thermal resistance. A mechanistic understanding of how the curing agent chemistry governs the transition from chemically controlled kinetics to diffusion-limited regimes, as well as the onset of vitrification is essential for the design of biobased epoxy networks.

Todorovic et al. (2021) demonstrated that ELO network formation involves complex, multistep mechanisms influenced by the steric hindrance of epoxy groups near the triglyceride core and by secondary reactions. They showed that curing agent selection strongly affects thermomechanical performance, with *T*_g_ ranging from 12 to 145 °C and storage moduli (*E*′) from 10 to 1940 MPa, depending on the chemical rigidity of the curing agent.^8^ Similarly, Menager et al. (2020) examined the curing of ELO with biobased dicarboxylic acids of different chain lengths (succinic, suberic, and sebacic acids) and demonstrated a direct correlation between curing agent structure, curing kinetics, and final properties. Short-chain succinic acid promoted higher reaction rates, greater cross-link density, and superior thermomechanical performance (*T*_g_ ≈ 20 °C; *E*′ ≈ 1.7 GPa), whereas longer-chain suberic and sebacic acids yielded more flexible networks with lower cross-link density (*T*_g_ ≈ 4–6 °C; *E*′ ≈ 1.1–1.4 GPa).^12^ These findings underscore how curing agent chemistry enables precise tuning of stiffness, thermal stability, and elasticity in ELO-based epoxy systems.

Since epoxy resins derived from renewable precursors, such as LO, typically exhibit complex, multistep curing mechanisms that depend strongly on the nature of the curing agent,^13–15^ a rigorous kinetic analysis is essential to understand these processes. In this context, advanced approaches combining isoconversional methods applied to nonisothermal and isothermal differential scanning calorimetry (DSC) data with spectroscopic monitoring by Fourier-transform infrared spectroscopy (FTIR) allow reaction profiles to be resolved without assuming predefined kinetic models.^16^ The construction of time–temperature–transformation (TTT) diagrams further enables the identification of distinct kinetic regimes and the simultaneous correlation of temperature, time, vitrification, and diffusion-controlled limitations, offering insights that cannot be captured by kinetic parameters alone. By revealing how curing-agent chemistry governs vitrification behavior, network architecture, and thermomechanical performance, this approach establishes TTT diagrams as a key tool for process control and rational design of high-performance, sustainable epoxy thermosets tailored to specific application and processing requirements.

This study investigates the curing behavior of ELO using two chemically distinct curing systems: methyltetrahydrophthalic anhydride activated with benzyltriethylammonium chloride and a biobased aliphatic diamine. By combining isothermal and nonisothermal kinetic analyses with complementary characterization techniques, DSC, attenuated-total-reflectance FTIR (ATR-FTIR) spectroscopy, isoconversional methods, rheological monitoring, dynamic mechanical analysis, and TTT diagrams, this study aims at bringing a mechanistic and quantitative comparison of curing pathways, kinetic regimes, and structure–property relationships in these ELO-based epoxy networks.

## Experimental Section

### Materials

The linseed oil (LO), with unsaturation content 6.25 per molecule evaluated by nuclear magnetic resonance (^1^H NMR) spectroscopy, was purchased from Sigma-Aldrich. Its fatty acid composition was determined according to the methodology proposed by Barison et al. (2010) by ^1^H NMR, was (%) oleic = 29, linoleic = 13, linolenic = 51, and saturated fatty acids = 9. However, a value higher than 100% was observed in total fatty acid concentration, due to free fatty acids presented.^17^ Oxone was purchased from Sigma-Aldrich, and acetone, ethyl acetate, sodium chloride, magnesium sulfate, and sodium bicarbonate were purchased from Êxodo Científica. As curing agents, an anhydride type, Listestone 2131H, composed of methyltetrahydrophthalic anhydride, containing 1–4% benzyltriethylammonium chloride, a quaternary ammonium compound, as an initiator, donated by Olin Corporation; and an amine type, Priamine 1071, a liquid diamine at room temperature, with an active hydrogen equivalent weight (AHEW) of 140 g eq^–1^, donated by Cargill BioIndustrial Inc. All reagents were used as received without further purification. Structures of reactants are described in Figure 1.

**Figure 1:**
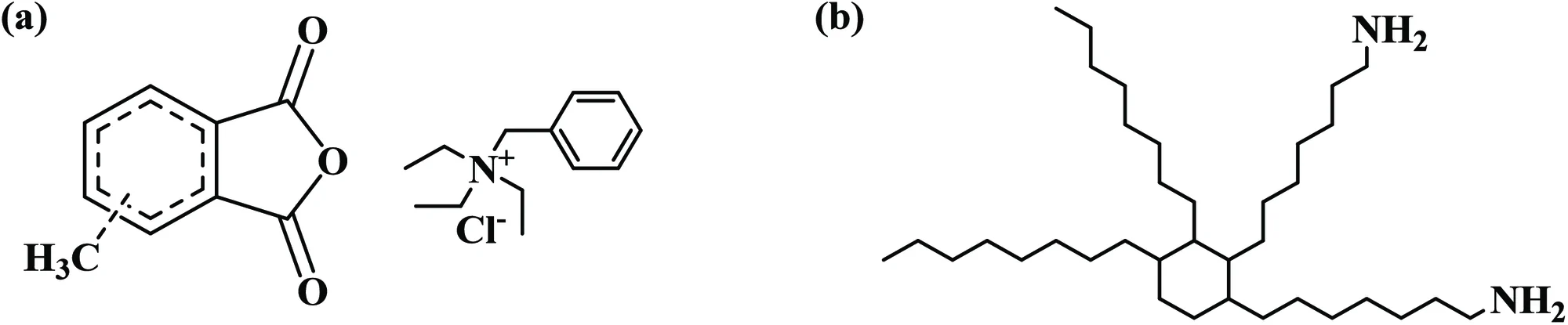
Chemical structures of the curing agents: (a) anhydride and initiator; (b) amine.

### Synthesis of ELO

The LO was epoxidized using the dimethyldioxirane intermediate route (Shi epoxidation), as described in the literature for vegetable oils.^18^ After a series of optimization experiments on the reaction parameters, the epoxidation of LO was carried out in a Büchiglasuster BMD 300 pilot reactor, with a capacity of two liters, equipped with an agitation system and temperature control. For the reaction, LO (24 g, 0.03 mol) was dissolved in acetone (800 mL, 632 g), and the mixture was transferred to the reactor along with sodium bicarbonate (43 g, 0.51 mol). Then, an aqueous solution of KHSO_5_·0.5KHSO_4_·0.5K_2_SO_4_ known by its commercial name Oxone (0.46 mol·L^–1^) was slowly added to the reaction, which was kept under constant stirring (500 rpm) and temperature control (not exceeding 40 °C). After approximately 5 h, when the reaction was complete, the mixture was transferred to a separatory funnel. The aqueous phase was then extracted with ethyl acetate and treated with small portions of a saturated sodium chloride solution to prevent emulsion formation. After extracting the organic phase, anhydrous magnesium sulfate was added to remove any remaining water. The organic solvent was then evaporated using a rotary evaporator for approximately 40 min (40 °C, 240 mbar) and subsequently placed in a reduced-pressure system for 8 h (room temperature, >10 mbar) to completely remove the solvent.

To determine the conversion degree of the unsaturated bonds to epoxy groups, the sample was analyzed by ^1^H NMR on a Bruker Avance 400 (9.39 T) using deuterated chloroform (CDCl_3_) as the solvent. Fourier transform infrared spectroscopy with attenuated total reflectance (ATR-FTIR) was also used to characterize ELO. The FTIR spectra were obtained on a Perkin-Elmer Spectrum 1000 instrument, in the range of 4000–500 cm^–1^, with 32 scans and a resolution of 4 cm^–1^.

### Cure Kinetics and Thermal Analysis

In this study, two formulations using ELO were investigated: with an anhydride-based curing agent (ELO-AD) and with an amine-based curing agent (ELO-AM). To ensure the consistency and comparability of the kinetic analyses, all formulations were prepared from the same homogeneous ELO batch synthesized under controlled conditions. For each formulation, three different epoxy-to-curing agent reactive group molar ratios were investigated and compared with the stoichiometric composition.

ATR-FTIR was performed for kinetic monitoring using a Perkin-Elmer Spectrum 1000 spectrophotometer over 4000–500 cm^–1^, with 32 scans and a resolution of 4 cm^–1^. Spectra were collected every 2 h during nonisothermal curing protocols specific to each formulation: for ELO-AD, the curing cycle was 130, 160, and 180 °C; for ELO-AM, sequential steps at 120, 140, and 160 °C were used. Both were cured for 12 h, based on the DSC analysis of each formulation.

DSC analyses were performed using a TA Instruments Q-20 calorimeter, with approximately 10 mg of uncured mixture sealed in hermetic aluminum pans. Nonisothermal DSC of the samples was performed at heating rates of 2, 5, 10, 15, and 20 °C·min^–1^. The DSC curve obtained at 10 °C·min^–1^ was used as a reference to assist in deconvoluting overlapping exothermic peaks, which were fitted with independent Gaussian functions. The degree of conversion (α) at each heating rate was calculated from the partial curing enthalpy (Δ*H*), proportional to the area under the exothermic peak, according to eq 1, where d*H*/d*T* represents the instantaneous heat flow, and *T*_0_ and *T*_∞_ correspond to the initial and final temperatures of the curing process, respectively.

1 $$\alpha \left(T\right)=\frac{\int_{T_{0}}^{T}\left(\frac{dH}{dT}\right)\;dT}{\int_{T_{0}}^{T_{\infty }}\left(\frac{dH}{dT}\right)\;dT}$$

Isothermal DSC analyses were performed by heating the samples at 200 °C·min^–1^ to 80, 100, 120, and 140 °C for the ELO-AD formulation, and 90, 110, 130, and 150 °C for the ELO-AM formulation. Each temperature was maintained for 7.5, 15, 30, 60, and 120 min. After the isothermal stage, the samples were cooled to −80 °C and subsequently reheated at 20 °C·min^–1^ to 250 °C for ELO-AD and 350 °C for ELO-AM. α­(*t*) was calculated according to eq 2, where Δ*H*_*t*_ is the enthalpy released up to time *t*, and Δ*H*_tot_ is the total reaction enthalpy.

2 $$\alpha \left(t\right)=\frac{{\Delta H}_{t}}{{\Delta H}_{\mathrm{tot}}}$$

To more comprehensively monitor the curing evolution, both formulations were investigated through isothermal and nonisothermal conditions. In systems based on ELO, the curing evolution can vary significantly depending on the type of curing agent. Due to the differences in the reaction mechanisms of the curing agents evaluated in this study, a more detailed analysis was required, as both may exhibit multiple reaction stages. The identification of these stages was carried out by evaluating the activation energy–conversion curve, *E*_a_–α­(*T*), and the derivative of the DSC curve, using the Friedman^19^ and Vyazovkin^20^ models, as described by eqs 3–5.

3 $$\ln{\left(\frac{\mathrm{d\alpha}}{dt}\right)}_{\alpha ,i}=\ln f\left(\alpha \right)+A-\frac{E_{\alpha }}{RT_{\alpha ,i}}$$

4 $$\Phi \left(E_{\alpha }\right)=\overset{n}{\underset{i=1}{\sum }}\overset{n}{\underset{j\neq i}{\sum }}\frac{J\left[E_{\alpha },T_{i}\left(t_{\alpha }\right)\right]}{{[E}_{\alpha },T_{j}\left(t_{\alpha }\right)]}$$

5 $$J\left[E_{\alpha },T\left(t_{\alpha }\right)\right]=\int_{t_{\alpha }-\Delta \alpha }^{t_{\alpha }}\exp\left[\frac{-E_{\alpha }}{RT\left(t\right)}\right]\;dt$$

where α is the extent of conversion, *t* is the time, *T* is the absolute temperature, and *R* is the universal gas constant.

The relationship between the glass transition temperature and the conversion (α) was obtained during the isothermal curing process. Equation 6 of Di Benedetto^21^ was applied, in which *T*_g0_ is the glass transition temperature of the uncured system (α = 0), *T*_g∞_ refers to the glass transition temperature of the fully cured system (α = 1), and λ corresponds to the cross-linking fitting parameter.

6 $$\frac{T_{g}-T_{\mathrm{g\infty}}}{T_{\mathrm{g\infty}}-T_{g0}}=\frac{\lambda \alpha }{1-\left(1-\lambda \right)\alpha }$$

In the kinetic modeling, the curing process was described using a sequential reaction scheme.^22^ In the first stage, curing was modeled as a first-order reaction, representing the initiation step in which reactive species are generated and the reaction rate is primarily temperature-dependent, with no significant autocatalysis. In the second stage, the main propagation of the epoxy network was described using the Kamal–Sourour autocatalytic model, which accounts for the acceleration of epoxy ring-opening reactions promoted by hydroxyl anion formed during curing. This stage dominates at intermediate degrees of conversion and is responsible for the main heat release observed in DSC. At high degrees of conversion, the formation of a cross-linked network restricts molecular mobility and leads to vitrification, limiting the reaction rate to diffusion rather than chemical reactivity. To capture this effect, diffusion control was incorporated via a Rabinowitch-type formulation that couples the intrinsic chemical reaction rate with a diffusion-controlled rate constant, enabling a smooth transition from chemically controlled to diffusion-controlled kinetics and preventing overestimation of reaction rates at high conversion. The diffusion-control parameters were simultaneously optimized with the Kamal–Sourour kinetic parameters through a global fitting procedure using both isothermal and nonisothermal DSC data. This global approach provides a consistent description of the curing kinetics over the entire conversion range and yields a single parameter set constrained by multiple experimental conditions, which was subsequently used to construct the TTT diagrams.^23–26^ All kinetic parameters were determined by global fitting of isothermal and nonisothermal DSC data using NETZSCH Kinetics Neo software.^27^

Thermogravimetric analysis (TGA) was performed on a TA Instruments Q50 instrument under an inert nitrogen atmosphere at 60 mL·min^–1^, with a heating rate of 10 °C·min^–1^, from room temperature to 900 °C.

### Rheology, Solid Viscoelastic, Mechanical, and Swelling Behavior

Rheological analysis of both systems was performed using formulations prepared from the same homogeneous ELO batch employed throughout this study, in an Anton Paar MCR 101 rheometer using a 50 mm parallel plate geometry with a 1 mm gap at 120, 130, and 140 °C, at a shear rate of 1 s^–1^. The viscosity data were modeled using an equation that integrates the resin flow description, based on the Andrade equation, with the thermal dependence expressed by the Arrhenius equation, where η_0_ is the viscosity of the uncured system at temperature *T*, *k*_η_ is the curing process advancement rate at isothermal temperature, and *t* is the curing time (eq 7).^28^

7 $$\ln\left[\eta {\left(t\right)}_{T}\right]=\ln\left(\eta_{0}\right)+k_{\eta }t$$

Solid viscoelastic measurements were conducted using dynamic mechanical analysis on a TA Instruments DMA Q850 operated in tension mode, in accordance with ASTM D5026-23. The experiments were carried out within the linear viscoelastic region, at an amplitude of 1%, over the temperature range from −80 to 150 °C at 2 °C·min^–1^, and at frequencies of 1, 5, 10, 50, and 100 Hz. Prismatic specimens (dimensions: 35 mm × 10 mm × 3.2 mm) were used. The cross-link density (υ) was calculated according to eq 8,^29^ where *E*′ is the storage modulus in the rubbery region, *R* is the universal gas constant, and *T* is the absolute temperature. The (*E*′) values were obtained from the DMA curves at 1 Hz, using a temperature within the rubbery region of each formulation: 125 °C for ELO-AD and 75 °C for ELO-AM.

8 $$\upsilon =\frac{{E_{r}}^{\prime }}{3RT}$$

Tensile tests were performed in accordance with ASTM D638-22 using an INSTRON 3382 machine and test speeds of 10 and 20 mm·min^–1^ for ELO-AD and ELO-AM, respectively. For these tests, a minimum of five samples per formulation were analyzed.

The swelling experiments were conducted with three specimens of approximately 1 g of cured resins for each formulation. The samples were immersed in toluene at 30 °C for 48 h. The degree of swelling (*Q*) was calculated as the mass ratio of the swollen to the dry sample using eq 9.^29^

9 $$Q=\frac{m_{s}-m_{0}}{m_{0}}$$

where *m*_s_ and *m*_0_ are the masses of the specimens after and before swelling, respectively.

Gel content was determined according to an adaptation of ASTM D2765-16. Approximately 0.3 g of each formulation was enclosed in a wire mesh envelope and extracted in boiling toluene for 8 h. After extraction, the samples were dried at 60 °C for 24 h and weighed to determine the insoluble gel fraction.

## Results and Discussion

### Synthesis of ELO

The synthesis of ELO was adapted from the procedures reported by Setien et al., applying their methodology to LO with specific modifications.^18^ The chemical structure of ELO was characterized by ^1^H NMR and FTIR spectroscopy. In the ^1^H NMR spectra (Figure 2a), the signal at 4.33 ppm corresponds to the methylene protons and the signal at 5.30 ppm to the methinic proton of the triacylglycerol backbone. The disappearance of signals in the 5.44 ppm region, attributed to the vinylic protons of unsaturated fatty acids, together with the appearance of signals at 2.86 and 3.04 ppm, assigned to epoxy protons, indicates the conversion of C=C double bonds into oxirane rings. From that, an average epoxidation degree of 5.54 epoxy groups per ELO molecule was determined, corresponding to a average conversion yield of 92%, indicating high reaction selectivity.

**Figure 2:**
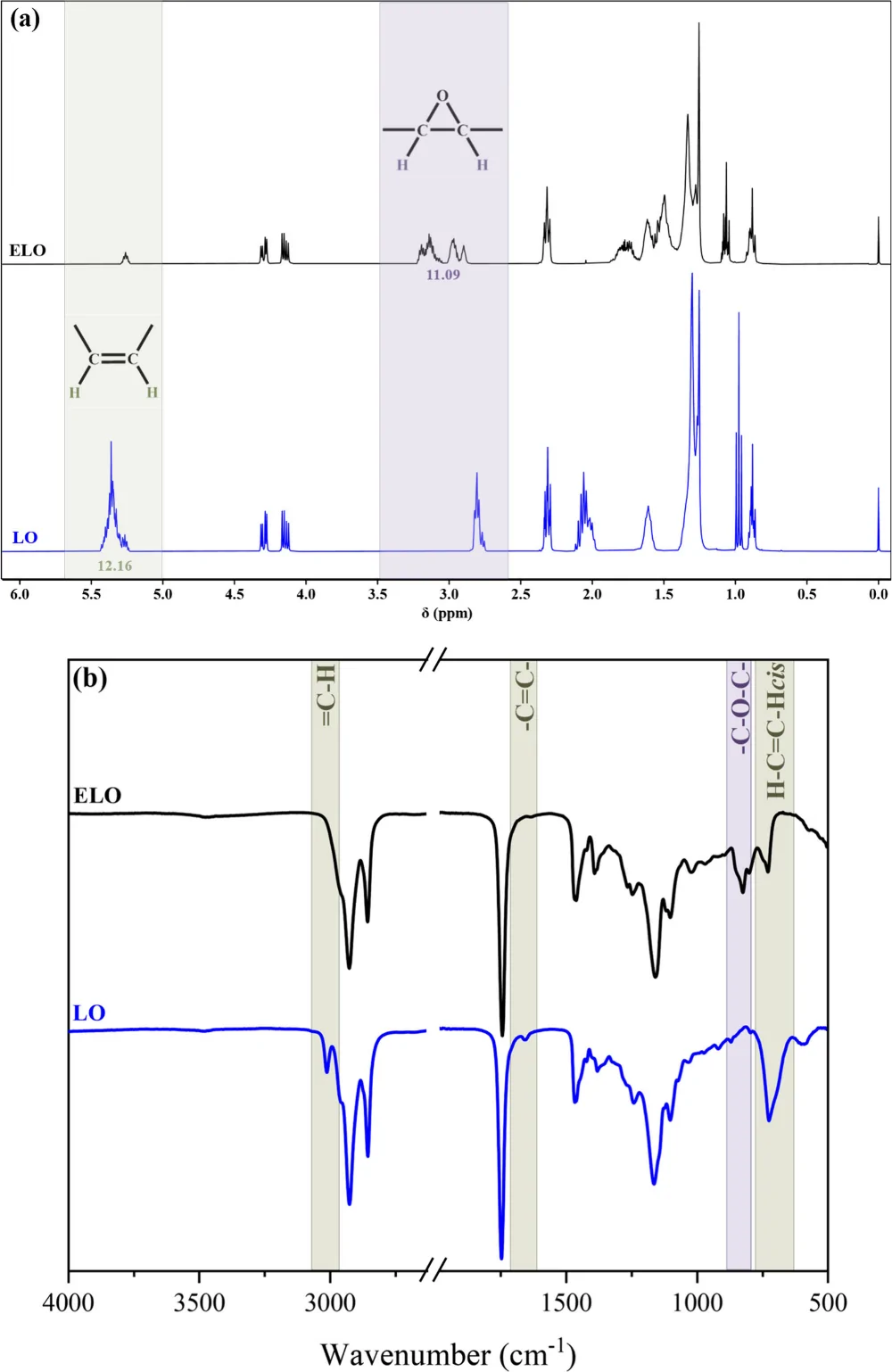
(a) ^1^H NMR spectra (CDCl_3_, 400 MHz) and (b) ATR-FTIR spectra of LO and ELO.

The FTIR spectrum of ELO (Figure 2b) further supports the successful epoxidation. Characteristic absorption bands associated with unsaturated bonds at 3014 cm^–1^ (=C–H), 1656 cm^–1^ (−C=C−), and 718 cm^–1^ (H–C=C–H*cis*) are absent after epoxidation, confirming the consumption of double bonds, in agreement with previous reports.^30^ In addition, the appearance of the band at 830 cm^–1^, assigned to the −C–O–C– stretching vibration of epoxy groups, provides clear evidence for the formation of oxirane functionalities in the ELO structure.

### Adjustment of the Resin Formulations

DSC was first used to evaluate curing parameters (*T*_p_, α, and *T*_g_) of formulations prepared with both curing agents (Figure 3 and Table 1). The ELO-AD systems exhibited two exothermic peaks, whereas ELO-AM showed multiple overlapping peaks, indicating distinct curing mechanisms. For anhydride-cured formulations, decreasing the anhydride-to-epoxy ratio increased *T*_g_ from 10 to 35 °C (Figure 3b). The ELO-AD (1:0.8) formulation showed the highest *T*_g_, *T*_p_, and conversion (α = 0.73), indicating a higher cross-link density, whereas the ELO-AD (1:1.5) formulation exhibited lower *T*_g_ and conversion (α = 0.66), likely due to unreacted epoxy groups. Reduced performance at stoichiometric conditions has been attributed to steric hindrance of epoxy groups near the triglyceride core, requiring a slight excess of epoxy for effective network formation.^8,9^

**Figure 3:**
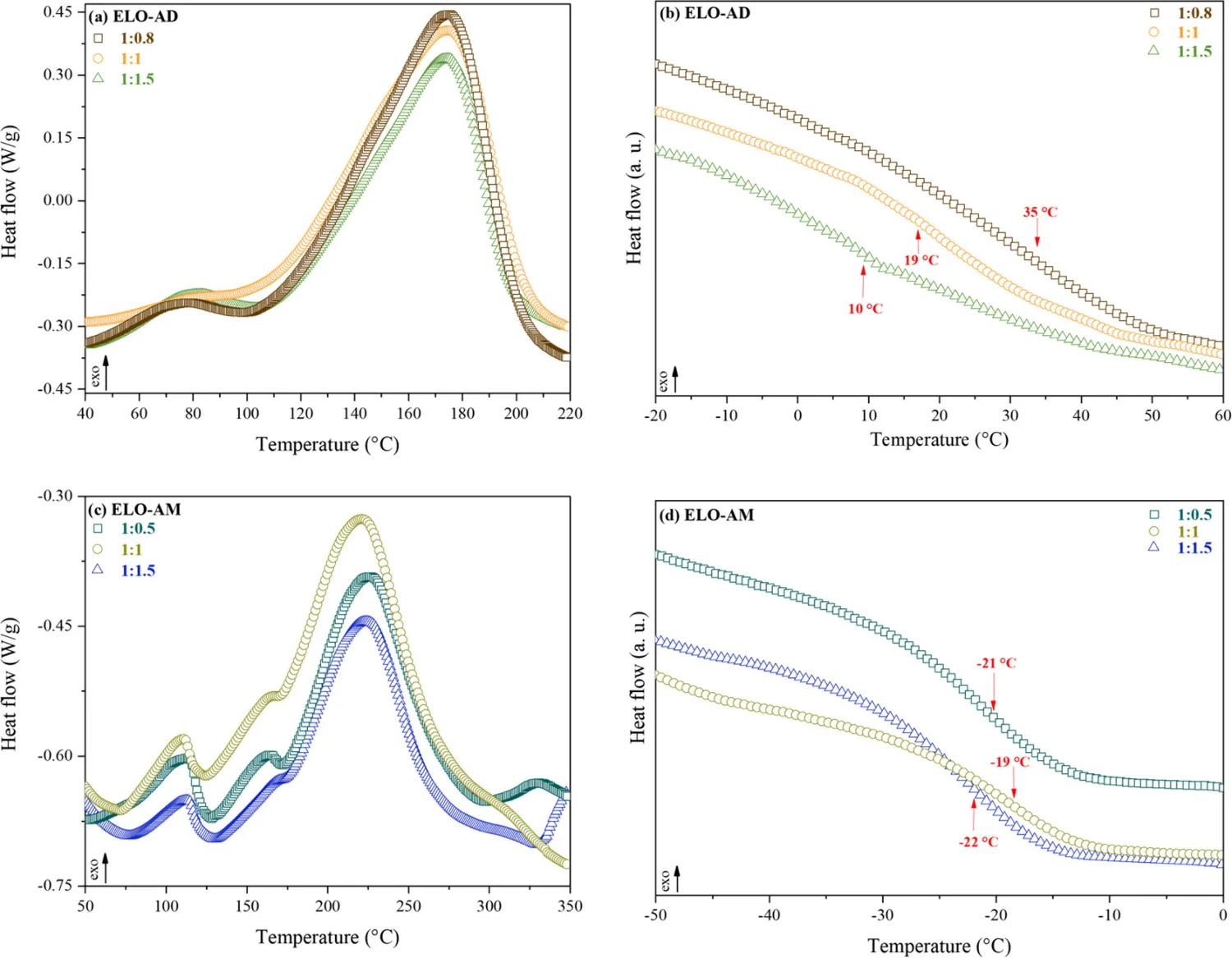
DSC curves of the ELO-AD: (a) curing peaks and (b) *T*_g_ curves. DSC curves of the ELO-AM: (c) curing peaks and (d) *T*_g_ curves.

**Table 1:** Effect of the Molar Ratio between Epoxy Resin (E) and Curing Agent (CA) (Reactive Groups)

Formulation	Molar ratio E:CA	α_p_	*T*_g_ (°C)	*T*_p_ (°C)
ELO-AD	1:0.8	0.73	35	176
	1:1	0.70	19	174
	1:1.5	0.66	10	173
ELO-AM	1:0.5	0.60	–21	225
	1:1	0.61	–19	226
	1:1.5	0.55	–22	224

In contrast, the amine-cured formulations displayed only minor variations in *T*_g_ (−21 to −19 °C), with a slight decrease at excess amine content (−22 °C for 1:1.5), consistent with lower conversion (α = 0.55). *T*_p_ values remained nearly constant (224–226 °C), indicating similar reaction onset across compositions, while final conversion and network structure varied with curing agent content.^13^ Formulations with the highest *T*_g_ and α were selected for kinetic analysis to compare the curing mechanisms.

## Curing Reactions

Representative FTIR spectra of the ELO-AD immediately after mixing and throughout the curing are shown in Figure 4. An increase in the broad absorption band at 3600–3300 cm^–1^, attributed to −O–H stretching, together with a decrease in the band at 3050 cm^–1^ associated with epoxy ring vibrations, indicates progressive epoxy ring opening.^9^ Concurrently, the growth of aliphatic −C–H stretching bands in the 3000–2850 cm^–1^ region reflects network formation through epoxy–anhydride reactions.^8^

**Figure 4:**
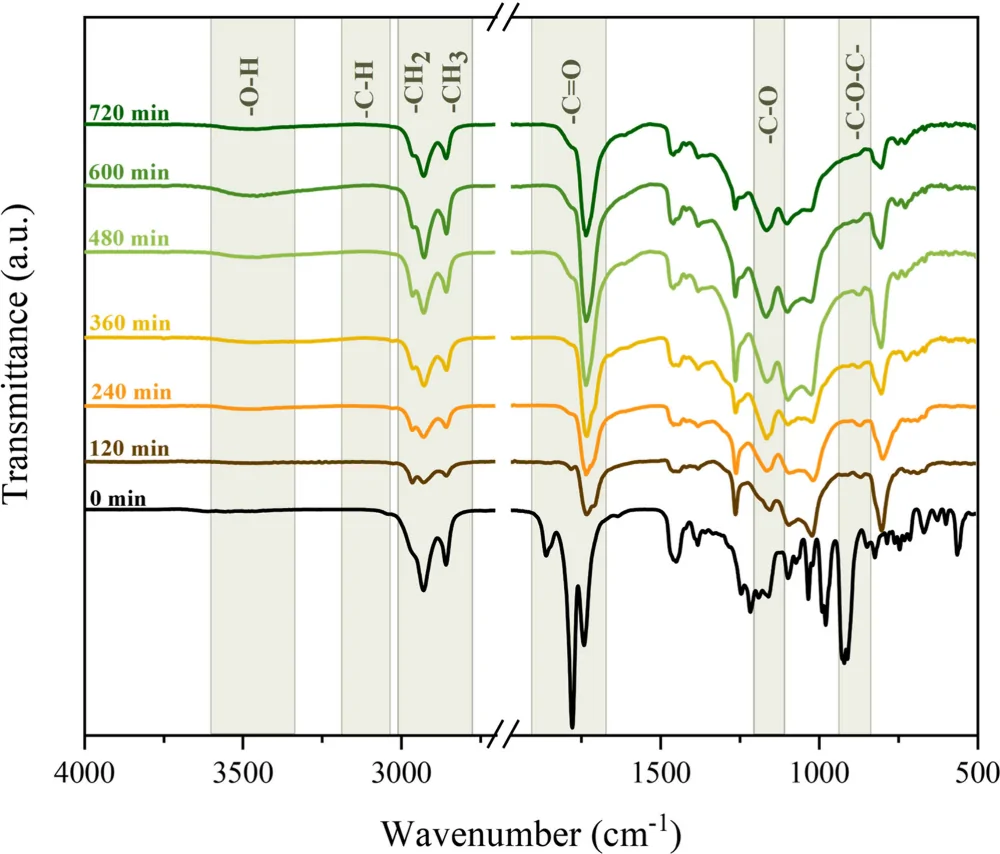
ATR-FTIR spectra of the ELO-AD throughout the curing process.

The gradual reduction of the anhydride carbonyl bands at 1860 and 1780 cm^–1^, accompanied by an increase in the ester carbonyl band at 1728 cm^–1^ and in the −C–O stretching bands at 1157 and 1148 cm^–1^, confirms that cross-linking proceeds predominantly via esterification, with a secondary contribution from etherification reactions.^31^ In parallel, the progressive decrease of the epoxy-related bands (−C–O–C−) at 915 and 830 cm^–1^ further evidence epoxy ring-opening reactions during curing.^32^

The epoxy–anhydride polymerization proceeds via an anionic ring-opening mechanism, as widely reported in the literature.^33^ In the present system, curing is initiated by the *in situ* generation of a tertiary amine resulting from thermal activation of the initiator contained in the curing agent, producing triethylamine and benzyl chloride.^34^ At early stages of curing, zwitterionic active species are generated through nucleophilic interactions involving the tertiary amine (Scheme 1). Specifically, nucleophilic attack of the amine on the epoxy ring leads to the formation of alkoxide species (Reaction I), while interaction with the anhydride carbonyl generates carboxylate species (Reaction III).^14^ These intermediates subsequently propagate the network through esterification reactions between alkoxides and anhydrides (Reaction II) and between carboxylates and epoxy groups (Reaction IV).

**Scheme 1:**
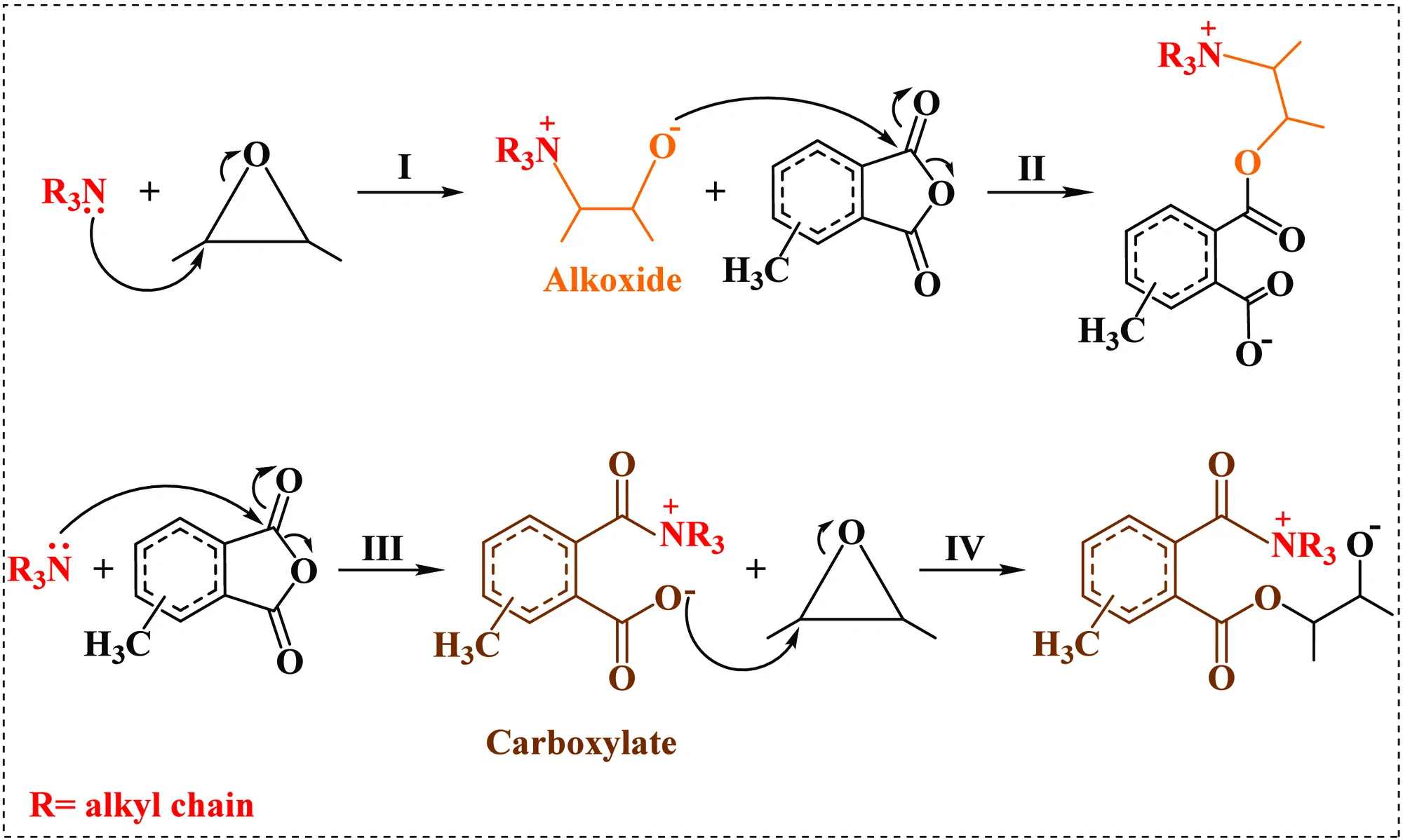
Schematic of the Curing Reaction of Epoxy–Anhydride Initiated by Tertiary Amine

Both epoxy and anhydride groups therefore participate throughout the curing process, occurring concurrently, as corroborated by FTIR analysis. However, the reaction between alkoxide species and anhydrides is kinetically favored over the carboxylate–epoxy reaction due to the higher electrophilicity of the anhydride, higher nucleophilicity of alkoxide, and the greater steric hindrance of the epoxy groups.^35^

Figure 5 shows the FTIR spectra of the ELO-AM before curing, immediately after mixing and during the curing process. As curing progressed, a gradual increase in the broad absorption band at 3600–3300 cm^–1^, attributed to −O–H stretching, together with the emergence of the −N–H stretching band at approximately 3350 cm^–1^, indicates epoxy ring-opening reactions accompanied by the formation of secondary amines through epoxy–amine addition.^36^ Concurrently, the growth of aliphatic −C–H stretching bands in the 3000–2850 cm^–1^ region reflects the advancement of network formation.

**Figure 5:**
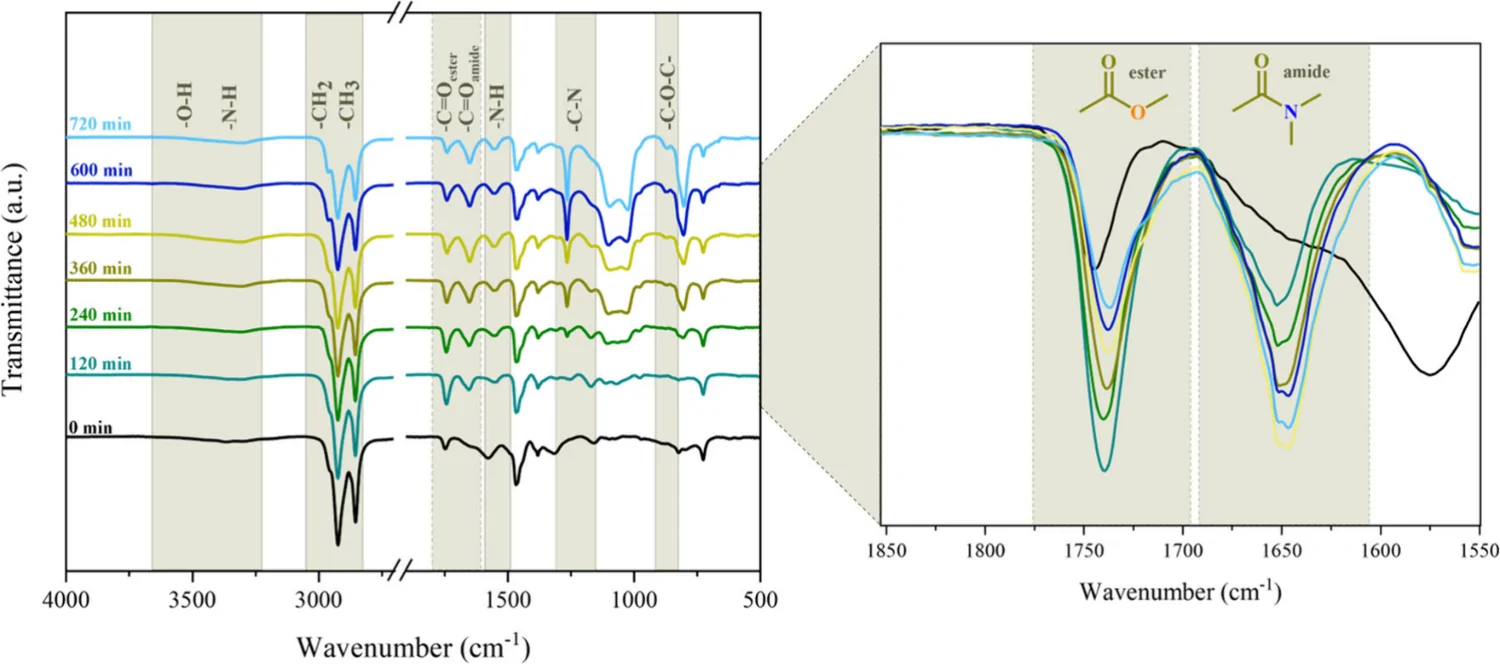
ATR-FTIR spectra of the ELO-AM throughout the curing process.

A progressive decrease in the ester carbonyl band at 1735 cm^–1^, associated with the triglyceride backbone, was also observed. Simultaneously, the appearance and growth of absorption bands at 1645 and 1550 cm^–1^, corresponding to the −C=O and −N–H stretching vibrations of amide groups, respectively, along with the increased intensity of −C–N stretching bands in the 1300–1220 cm^–1^ region, demonstrate that aminolysis reactions occur during curing.^13^ In parallel, the gradual reduction of epoxy-related bands at 915 and 830 cm^–1^ confirms the consumption of epoxy groups through cross-linking reactions, consistent with the proposed curing mechanism.^37^

During the curing of the ELO-AM, two concurrent reaction pathways were identified, as illustrated in Scheme 2. The primary pathway involves nucleophilic attack of primary amine groups on the less sterically hindered carbon of the epoxy ring, leading to epoxy ring opening and formation of hydroxyl groups adjacent to C–N bonds, with concomitant generation of secondary amines (Reaction I, Scheme 2).^38^ These secondary amines may further react with additional epoxy groups, yielding tertiary amines, as supported by FTIR analysis.

**Scheme 2:**
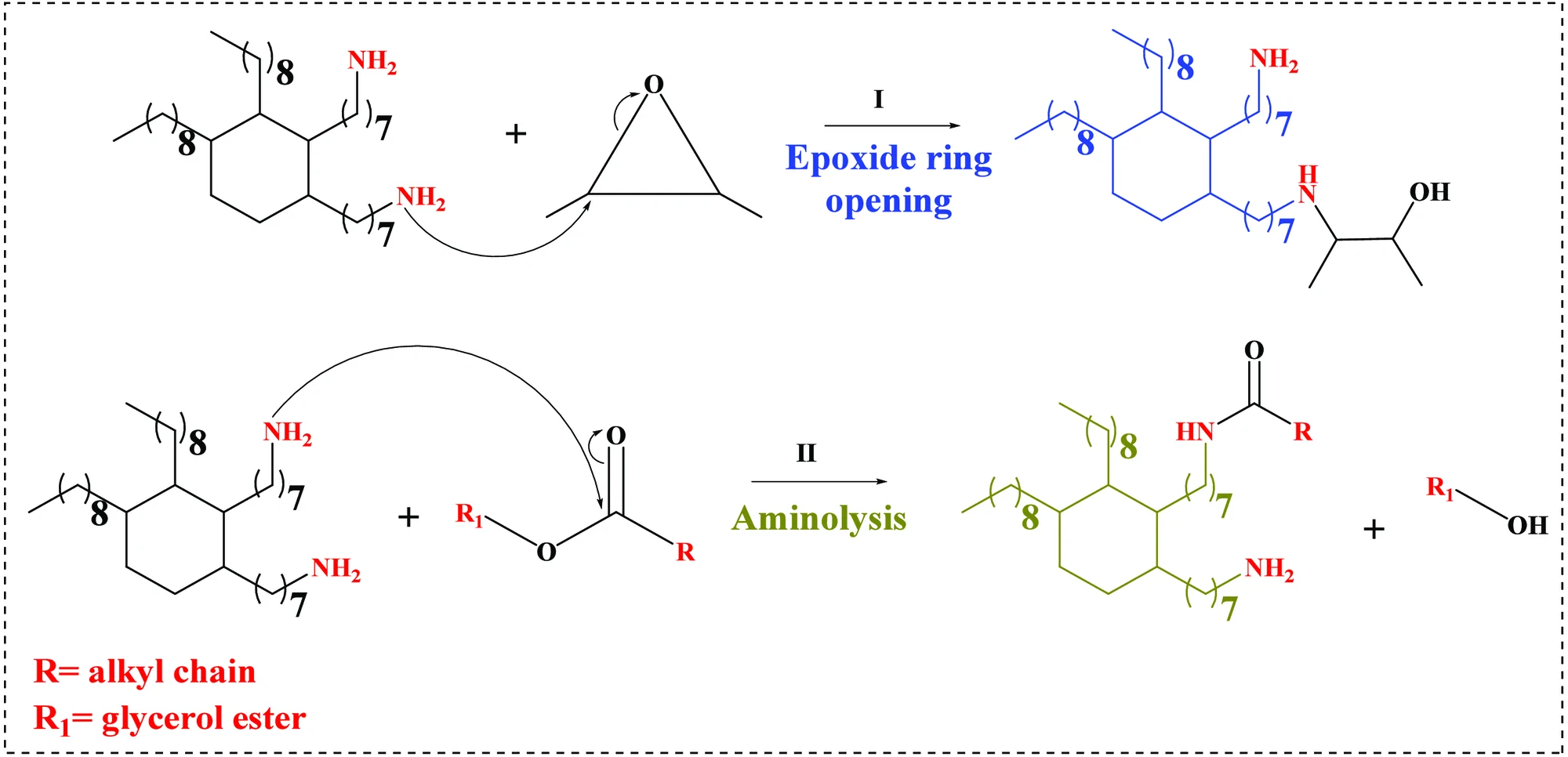
Schematic of the Curing Reaction of Epoxy–Diamine

In parallel, aminolysis reactions occur through nucleophilic attack of amine groups on the ester linkages of the triacylglyceride backbone of ELO, resulting in ester cleavage and formation of amide groups (Reaction II, Scheme 2). This secondary reaction pathway yields partial network degradation lowering *T*_g_ as observed at high amine content, particularly at the 1:1.5 molar ratio. The occurrence of aminolysis was corroborated by FTIR spectra, which show a reduction in ester-related absorption bands and the emergence of characteristic amide bands, consistent with previous reports.^39^

### Curing Kinetics by DSC and Thermal Analysis

The curing kinetics of ELO-AD and ELO-AM formulations were investigated by nonisothermal DSC (Figure 6). For both formulations, *T*_p_ increased with increasing heating rate, reflecting heat-transfer effects and reduced chain mobility during network formation. ELO-AD exhibited lower *T*_p_ values (142–188 °C; Figure 6a) and higher reaction enthalpies (Δ*H* = 297–345 J·g^–1^) than ELO-AM (*T*_p_ = 169–239 °C; Δ*H* = 178–242 J·g^–1^; Figure 6c), indicating faster curing kinetics and a higher extent of cross-linking for the anhydride-cured system.

**Figure 6:**
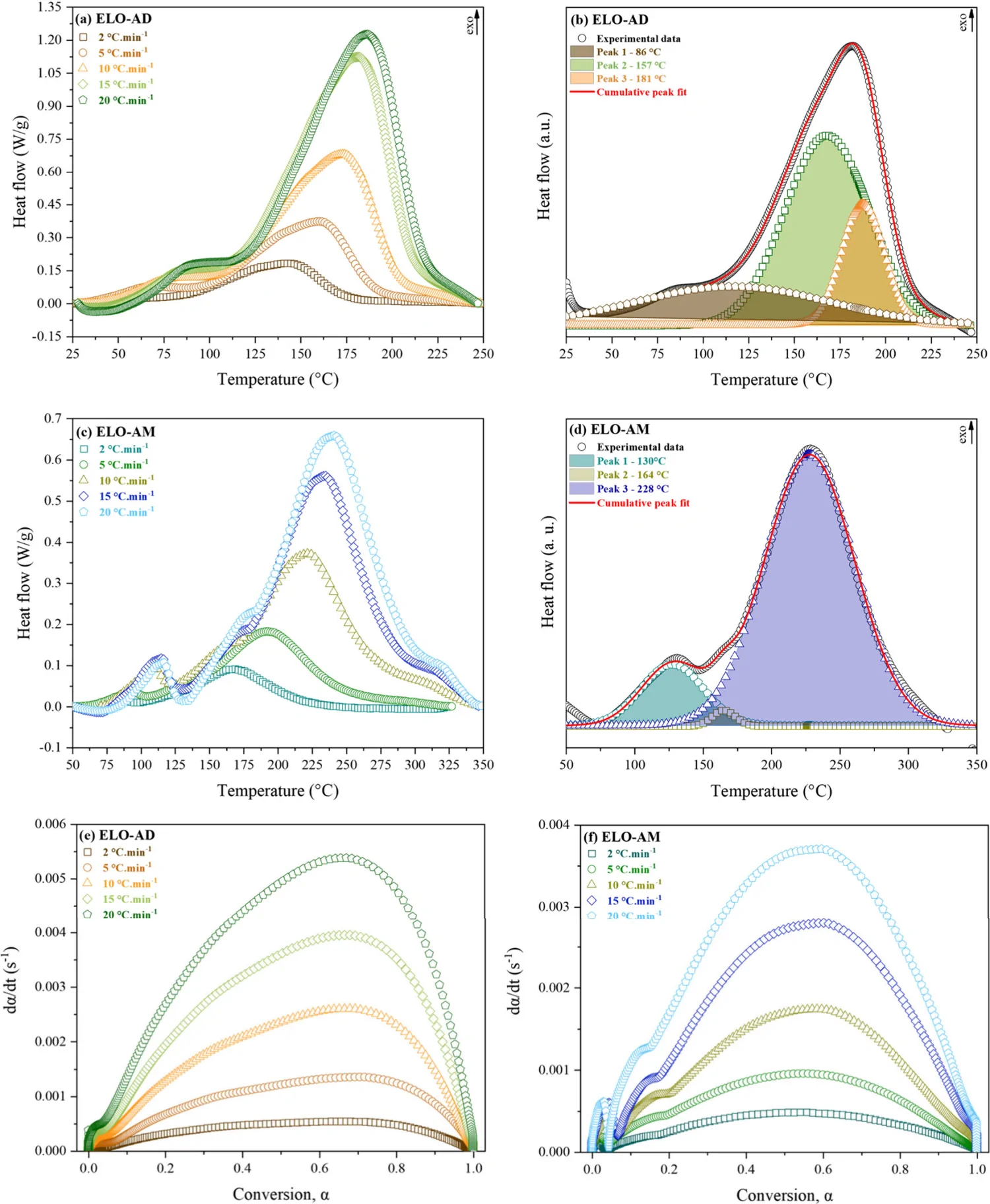
DSC curves of ELO-AD: (a) curing system with different heating rates and (b) deconvolution of data and fitted peaks. DSC curves of ELO-AM: (c) curing system with different heating rates and (d) deconvolution of data and fitted peaks. (e and f) Nonisothermal curing rate (dα/d*t*) as a function of α for ELO-AD and ELO-AM, respectively.

Both systems displayed broad and asymmetric DSC peaks, indicative of overlapping thermal events and multistep curing behavior arising from differences in epoxy accessibility and reactivity (Costa, Gerbase and Petzhold, 2011; Xavier et al., 2024). Peak deconvolution using independent Gaussian functions (*R* ^2^ > 0.99) revealed distinct curing stages (Figure 6b,d). For ELO-AD, the first peak (*T*_p_ ≈ 86 °C) is attributed to reaction initiation promoted by tertiary amine species, while subsequent peaks at approximately 157 and 181 °C correspond to the main cross-linking stages, consistent with epoxy–anhydride systems initiated by tertiary amines.^40^ In contrast, ELO-AM exhibited higher initiation and propagation temperatures, with peaks at ∼130, ∼164, and ∼228 °C, reflecting the coexistence of multiple reaction pathways characteristic of epoxy–amine systems.^39^ These features corroborate the multistep cross-linking mechanisms inferred from FTIR analysis. The degree of conversion at the peak heat flow (α_p_) differed significantly between the systems, reaching ∼0.65 for ELO-AD and ∼0.54 for ELO-AM, indicating that the maximum reaction rate occurs at more advanced conversion for the anhydride system. This behavior is characteristic of a more effective contribution of autocatalytic curing, whereas the lower α_p_ of ELO-AM reflects a more gradual network development and reduced thermal sensitivity.^41^ Minor variations in *T*_g_ with heating rate were observed in the second heating scan for both systems, likely due to incomplete equilibration at higher heating rates.^15^ The data of both formulations are summarized in Table 2.

**Table 2:** Nonisothermal Cure Parameters from DSC Analyses of ELO-AD and ELO-AM Formulations

		β (°C·min^–1^)				
Formulation		2	5	10	15	20
ELO-AD	*T*_p_ (°C)	142	159	174	183	186
	Δ*H*_tot_ (J·g^–1^)	345	313	297	316	267
	α_p_	0.66	0.66	0.65	0.66	0.64
	*T*_g_ (°C)	37	37	35	34	30
ELO-AM	*T*_p_ (°C)	180	193	209	228	231
	Δ*H*_tot_ (J·g^–1^)	178	201	226	235	242
	α_p_	0.71	0.55	0.46	0.52	0.47
	*T*_g_ (°C)	–20	–19	–19	–18	–23

Analysis of the curing rate as a function of conversion (dα/d*t* vs α; Figure 6e,f) further highlights these differences. ELO-AD exhibits three distinct rate maxima at α ≈ 0.03–0.05, 0.33–0.35, and 0.66–0.69, with an initial increase in reaction rate with α followed by a decline at high α due to diffusion control, a hallmark of autocatalytic systems. In contrast, ELO-AM shows rate maxima at lower conversions (α ≈ 0.03–0.05, 0.15–0.20, and 0.55–0.59), indicating a slower, more evenly distributed curing process and delayed network buildup. These kinetic features are fully consistent with the Kamal–Sourour autocatalytic model combined with diffusion control and underpin the subsequent *E*_a_(α) analysis and TTT diagram construction.

The results from the isothermal analyses for the ELO-AD and ELO-AM formulations are summarized in Tables S1 and S2, respectively. For the ELO-AD, isothermal DSC showed an intense initial reaction, followed by a gradual deceleration, with curing efficiency strongly dependent on temperature. At 120 and 140 °C, higher conversions were achieved within shorter times, with the reaction essentially completed after approximately 60 min at 120 °C and 30 min at 140 °C. These results indicate accelerated kinetics and more effective network formation at elevated temperatures. The Δ*H*_tot_ ranged from 214 to 353 J·g^–1^, reaching a maximum at 120 °C, consistent with extensive cross-linking. Accordingly, the *T*_g_ increased significantly during isothermal curing, from −7 to 50 °C, reflecting substantial advancement of the polymer network.

In contrast, the ELO-AM exhibited markedly slower isothermal curing behavior. At 90, 110, and 130 °C, low conversions were obtained, indicating inefficient curing under these conditions. At 150 °C, the reaction proceeded more gradually, with sustained heat flow over time, suggesting slower, yet continuous, network formation. Even at this higher temperature, the total curing enthalpy remained limited (77–144 J·g^–1^), well below that of the ELO-AD system. Correspondingly, *T*_g_ values remained low, varying from −58 to −25 °C after 120 min of curing, indicating restricted cross-linking and a less densely connected network. The isothermal results reinforce the conclusions drawn from nonisothermal DSC, confirming that the anhydride-cured system exhibits faster kinetics, higher conversion, and more effective network formation. In contrast, the amine-cured system shows slower curing, lower enthalpy release, and limited development of cross-link density.

Figure 7 presents the *E*_a_(α) profiles for the ELO-AD and ELO-AM formulations obtained by both isoconversional methods. The close agreement between the Friedman and Vyazovkin results across the entire conversion range confirms the robustness of the analysis and indicates that the observed trends reflect intrinsic kinetic behavior rather than numerical artifacts. For the ELO-AD, *E*_a_ increases sharply at low conversions (α < 0.1), then rises continuously to α ≈ 0.85, followed by a slight additional increase toward full conversion, spanning approximately 50–140 kJ·mol^–1^. These values are consistent with those reported for epoxy–anhydride systems initiated by tertiary amines.^15^ The strong conversion dependence of *E*_a_ reflects a progressive shift from chemically controlled kinetics to diffusion-limited curing, driven by rapid cross-link density buildup, reduced segmental mobility, and the onset of vitrification. This behavior also explains the presence of multiple overlapping exothermic peaks observed in nonisothermal DSC analyses.^15,42^

**Figure 7:**
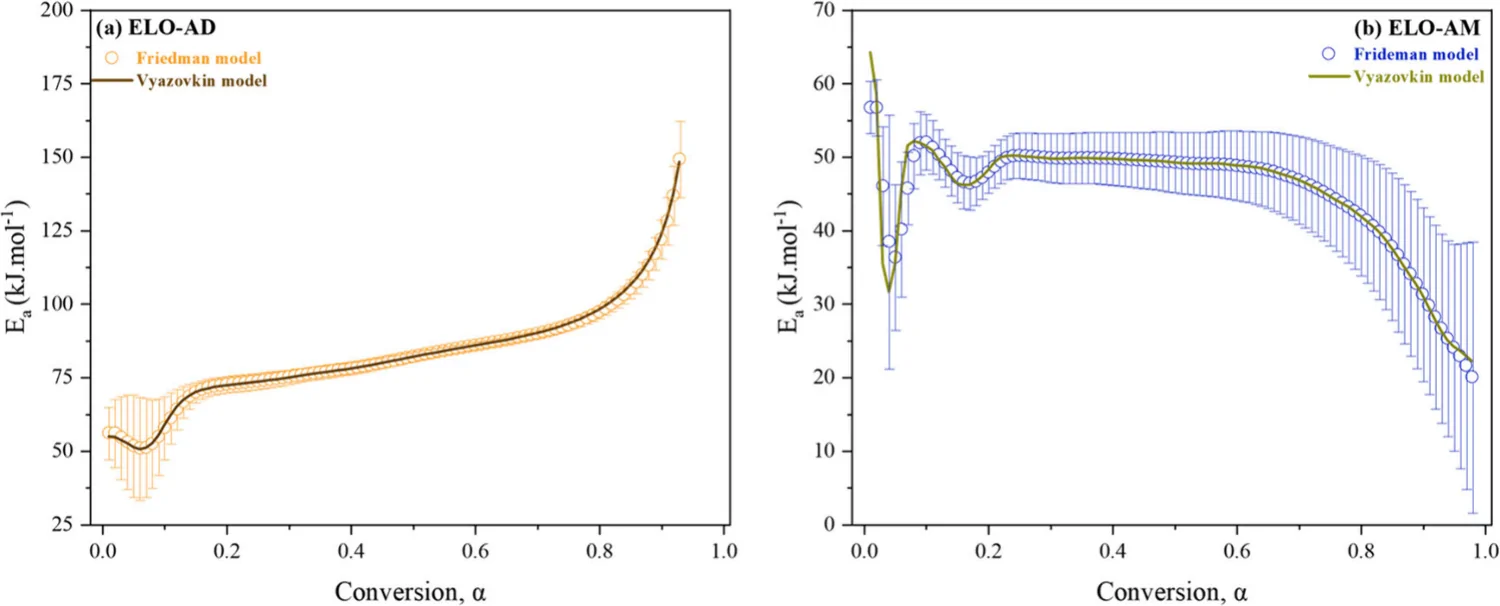
*E*_a_ vs α for the Friedman and Vyazovkin methods for (a) ELO-AD and (b) ELO-AM.

ELO-AM exhibits substantially lower activation energies, ranging from approximately 20 to 63 kJ·mol^–1^, with limited dependence on α. At low conversions (α < 0.1), the relatively low *E*_a_ indicates that the initial epoxy–amine addition proceeds with a modest apparent energy barrier. Between α ≈ 0.3 and 0.7, a broad *E*_a_ plateau around 50 kJ·mol^–1^ is observed, suggesting a stable propagation regime. At higher conversions (α > 0.7), *E*_a_ decreases toward ∼20 kJ·mol^–1^, indicating a transition to diffusion-controlled kinetics in which the apparent *E*_a_ is increasingly governed by mobility constraints and local rearrangements rather than intrinsic chemical barriers. Similar *E*_a_ ranges and trends have been reported for epoxy–amine systems undergoing gelation and vitrification.^43,44^ It should be noted that α ≈ 0.85 discussed for ELO-AD does not represent the maximum conversion achieved, but rather the conversion range associated with a change in the evolution of the apparent activation energy. The selected ELO-AD formulation (1:0.8) exhibited the highest *T*_g_ and conversion among the compositions investigated, while further optimization of curing temperature or postcuring time could potentially enhance the reaction of less-accessible epoxy groups.

Although the ELO-AM requires higher curing temperatures, as evidenced by nonisothermal DSC peak shifts, it shows consistently lower apparent activation energies than ELO-AD. This apparent contrast highlights the distinction between thermal requirements imposed by diffusion and vitrification effects and the intrinsic energetic barriers of the elementary reactions. Overall, the *E*_a_(α) trends confirm that ELO-AD curing is dominated by strongly conversion-dependent, autocatalytic-to-diffusion-controlled kinetics, whereas ELO-AM follows a more gradual curing pathway with lower apparent energy barriers but pronounced mobility control at advanced cure stages.

The evolution of the glass transition temperature with α for the ELO-AD and ELO-AM (Figure 8) was successfully described using the Di Benedetto equation, yielding excellent agreement with experimental data (*R* ^2^ > 0.99). The fitted parameters provide direct insight into the development of the network structure, cross-linking efficiency, and vitrification behavior for each system.

**Figure 8:**
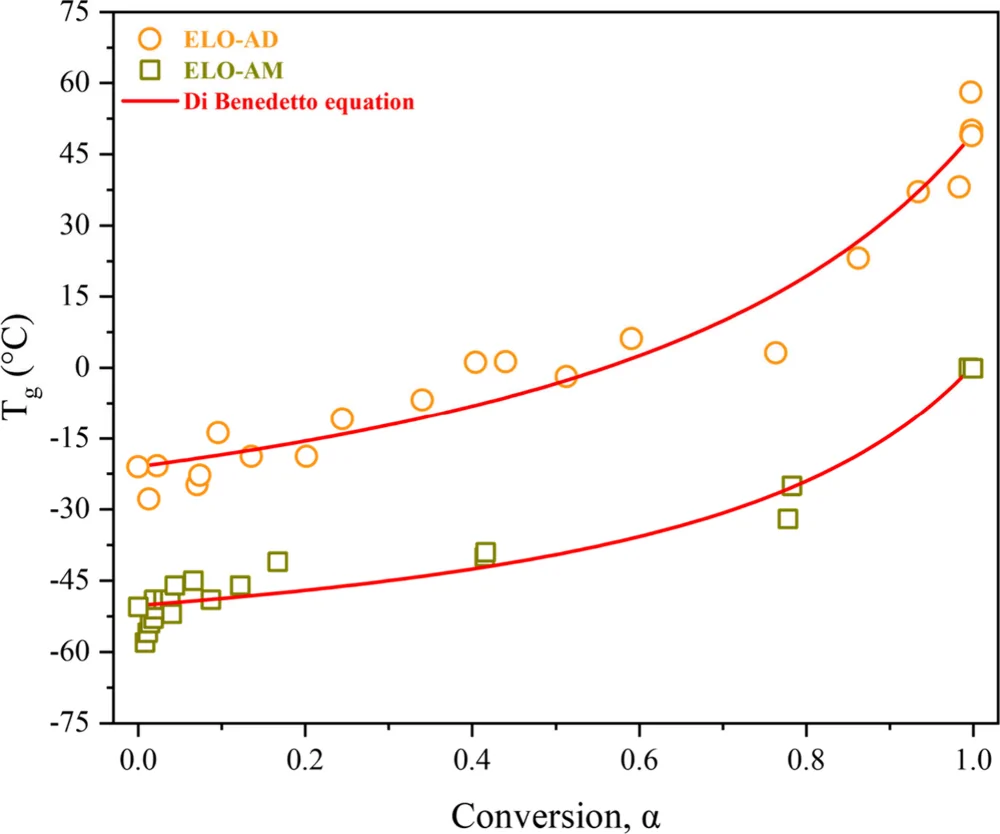
*T*_g_ as a function of α and Di Benedetto curve for the ELO-AD and ELO-AM formulations.

For the ELO-AD, the initial glass transition temperature (*T*_g0_ = −21.2 °C) reflects the relatively flexible nature of the uncured ELO. As curing proceeds, *T*_g_ increases sharply toward a final value of *T*_g∞_ ≈ 48.8 °C, indicating the formation of a densely cross-linked three-dimensional network. The relatively high *T*_g∞_ confirms efficient incorporation of the anhydride curing agent into the network and is consistent with the higher curing enthalpy and degree of conversion observed by DSC.

The λ parameter (λ = 0.336) provides key structural information, as it reflects how chemical conversion translates into restrictions on segmental mobility. The higher λ value for ELO-AD indicates strong coupling between conversion and network rigidity, meaning that relatively small increases in α lead to significant increases in *T*_g_. This behavior explains the early onset of vitrification observed in this system and supports the strong diffusion-controlled regime identified at high conversions through isoconversional *E*_a_(α) analysis. Consequently, in the TTT diagram, the vitrification line for ELO-AD is expected to occur at relatively short times and moderate temperatures, imposing kinetic limitations on further curing at advanced stages.

In contrast, the ELO-AM exhibits a markedly different *T*_g_(α) evolution. The very low initial *T*_g0_ (−50.5 °C) reflects the high chain mobility of the uncured epoxy–amine mixture. Even at full conversion, *T*_g∞_ remains close to −0.1 °C, indicating a substantially less rigid, less densely cross-linked network than ELO-AD. This low *T*_g∞_ is consistent with the lower curing enthalpy, the reduced α, and the occurrence of competing reactions, such as aminolysis, which disrupt the efficient network.

The lower λ value for ELO-AM (λ = 0.269) further indicates a weaker coupling between chemical conversion and structural stiffening. In this system, increases in α are reflected in more gradual changes in *T*_g_, indicating a curing process dominated by slower network organization and prolonged mobility of polymer segments. As a result, vitrification is significantly delayed, allowing the reaction to proceed over a wider time–temperature window. This behavior is consistent with the lower apparent activation energies observed in the isoconversional analysis and explains why higher curing temperatures are required to achieve meaningful conversion despite the lower intrinsic energy barriers of the elementary reactions.

The Di Benedetto parameters clearly distinguish the two curing mechanisms. The ELO-AD exhibits efficient cross-linking, strong conversion–mobility coupling, and early vitrification, leading to pronounced diffusion-controlled kinetics at high conversion. In contrast, the ELO-AM exhibits a more gradual *T*_g_ evolution, reduced network rigidity, and delayed vitrification, leading to extended curing times and a broader processing window. These differences directly underpin the distinct vitrification lines and kinetic domains observed in the corresponding TTT diagrams and validate the incorporation of diffusion control in the kinetic modeling of both systems.

Considering that both curing systems exhibit complex multistage reaction behavior, as evidenced by the nonisothermal DSC curves shown in Figure 6, a more detailed kinetic interpretation is required. The ELO resin contains epoxy groups with varying degrees of accessibility due to steric constraints and molecular architecture. This heterogeneity promotes sequential reactivity, in which epoxy groups located at more exposed regions of the molecule react preferentially at early stages, while sterically hindered or internally located epoxy groups participate predominantly at later stages of the curing process.

The nature of the curing agents further modulates this effect. Molecules with larger hydrodynamic volumes, such as the amine used as the AM curing agent, are expected to exhibit slower diffusion and reduced access to reactive sites, particularly as network formation progresses and molecular mobility decreases. To quantitatively assess these effects, hydrodynamic volumes were estimated from three-dimensional molecular conformations generated using RDKit with the ETKDG algorithm, followed by force-field optimization and calculation of van der Waals molecular volume and shape descriptors. An equivalent hydrodynamic radius was then obtained by applying a shape correction based on molecular asphericity. The resulting hydrodynamic volumes were 0.23 nm^3^ for the anhydride curing agent, 1.53 nm^3^ for the ELO resin, and 1.50 nm^3^ for the amine curing agent. These values indicate that, despite differences in chemical functionality, both curing agents interact with the ELO resin under diffusion-influenced conditions, leading to overlapping and sequential reaction events rather than a single, well-defined curing step.

Based on these considerations, the curing kinetics of the epoxy systems cured with anhydride and amine curing agents were analyzed by fitting different kinetic models directly to the DSC heat flow signal. The evaluated models included a two-step, sequentially ordered Kamal–Sourour model with diffusion control coupled with the Williams–Landel–Ferry (WLF) (A → B → C), a single-step Kamal–Sourour model, and an nth-order single-step model. The quality of the fits was assessed using the coefficient of determination (R^2^), the sum of deviation squares, the mean residual, and the number of degrees of freedom.

For the anhydride-cured system, the two-step Kamal–Sourour model provided an excellent description of the DSC signal, yielding an R^2^ of 0.990, a sum of deviation squares of 4.537, and a mean residual of 0.022, based on 4135 degrees of freedom. The single-step *n*th-order model yielded a slightly inferior fit (*R* ^2^ = 0.988), with a higher sum of squared deviations (5.556) and a mean residual of 0.024, despite a comparable number of degrees of freedom (4145). The single-step Kamal–Sourour model achieved a marginally higher *R* ^2^ value (0.991) and a lower sum of deviation squares (4.183), with a mean residual of 0.021 and 4142 degrees of freedom. Although its numerical performance is comparable to that of the two-step approach, the latter was selected for further analysis because it explicitly represents sequential reaction stages and diffusion-controlled effects, which are consistent with the heterogeneous accessibility of epoxy groups in the ELO resin.

A similar trend was observed for the amine-cured system. The two-step Kamal–Sourour model again exhibited the best overall performance, with an *R* ^2^ of 0.994, a sum of deviation squares of 0.846, and a mean residual of 0.009, supported by 4032 degrees of freedom. The single-step Kamal–Sourour model also provided a high-quality fit (*R* ^2^ = 0.992), with a sum of deviation squares of 1.053 and an identical mean residual of 0.009, based on 4045 degrees of freedom. In contrast, the single-step *n*th-order model showed the lowest fit quality among the tested approaches, with an R^2^ of 0.987, a sum of squared deviations of 1.722, and a mean residual of 0.013, despite a slightly higher number of degrees of freedom (4048). These results indicate that, although all models are statistically acceptable, Kamal-based formulations provide a more appropriate kinetic description than purely empirical nth-order models for capturing the diffusion-affected, multistage curing behavior of amine-cured epoxy systems.

Table 3 summarizes the kinetic parameters obtained from the two-step Kamal–Sourour model with diffusion control for the anhydride- and amine-cured ELO systems. In both systems, the second step (B → C) dominates the overall reaction, accounting for approximately 88–91% of the total reaction enthalpy, indicating that network consolidation and cross-linking occur predominantly at advanced conversion. The WLF parameters were nearly identical for both curing systems (*C*_1_ = 4.90–5.00 and *C*_2_ = 5.00 K), indicating a similar temperature dependence of segmental mobility.^23–26^ Therefore, the differences in curing behavior are primarily associated with the intrinsic reaction kinetics and the development of diffusion limitations rather than with differences in the WLF parameters.

**Table 3:** Sequential A → B → C Kinetic Model (Kamal–Sourour with Diffusion Control): Parameters of Fit for Anhydride- and Amine-Cured Systems

Parameter	Anhydride A → B	Anhydride B → C	Amine A → B	Amine B → C
*E*_a_1__ (kJ·mol^–1^)	40.00	45.17	19.00	26.14
log(*A*_1_) (s^–1^)	3.22	2.82	–2.50	–0.12
Reaction order, *n*	1.01	1.18	1.67	1.74
log(*A*_2_) (s^–1^)	0.86	6.00	3.34	6.33
Autocatalytic exponent, m	0.54	0.01	0.43	0.17
*E*_a_2__(kJ·mol^–1^)	40.83	94.04	21.92	78.65
log(*K*_diff_)	–4.05	5.00	9.44	5.00
*C*_1_ (diffusion)	4.90	5.00	5.00	5.00
*C*_2_ (K)	5.00	5.00	5.000	5.00
Contribution (Δ*H* fraction)	0.087	0.913	0.112	0.881

In the anhydride-cured system, both reaction steps exhibit relatively high activation energies (*E*_a_1__ = 40.0–45.2 kJ·mol^–1^), together with moderate reaction orders (*n* ≈ 1.0–1.2). The first step shows a clear autocatalytic contribution (*m* = 0.54), while the second step becomes essentially nonautocatalytic (m ≈ 0.01) and strongly diffusion-limited [log­(*K*_diff_) = 5.00]. As a consequence, curing progressively shifts from chemical control to diffusion control, leading to higher vitrification temperatures.

In contrast, the amine-cured system displays significantly lower activation energies in both steps (*E*_a_1__ = 19.0–26.1 kJ·mol^–1^), combined with higher reaction orders (*n* ≈ 1.7). Although epoxy–amine reactions are intrinsically more reactive, the progressive development of the cross-linked network restricts access to the remaining, sterically hindered epoxy groups, increasing the contribution of diffusion control at advanced conversion. Consequently, longer characteristic curing times are observed in the TTT diagram despite the lower activation energies. The anhydride system is therefore characterized by more thermally activated curing, whereas the amine system combines higher intrinsic chemical reactivity with stronger diffusion limitations at advanced conversion.

The differences in curing behavior between the anhydride- and amine-cured ELO systems arise from fundamental molecular factors related to reaction mechanism, molecular mobility, and network architecture. The ELO resin contains epoxy groups with heterogeneous steric accessibility along its bulky aliphatic backbone, inherently favoring sequential rather than concerted reactions. In the anhydride-cured system, epoxy–anhydride ring-opening and esterification require higher activation energies and progressively form an ester-rich cross-linked network. In contrast, epoxy–amine reactions proceed with lower activation barriers and generate C–N linkages and secondary hydroxyl groups. As curing progresses, network formation increasingly restricts the accessibility of residual epoxy groups, favoring diffusion-controlled behavior and competing reactions such as aminolysis. These molecular-level differences account for the distinct curing kinetics observed experimentally and provide the basis for the subsequent analysis of the TTT diagrams.

In contrast, epoxy–amine reactions proceed with lower activation barriers and generate flexible C–N linkages and secondary hydroxyl groups. Although chemically faster, this results in a more compliant network with a lower *T*_g_, leading to earlier vitrification under isothermal conditions. As a consequence, diffusion limitations dominate at advanced conversion, restricting access to sterically hindered epoxy groups favoring the aminolysis reaction. Thus, the amine-cured system becomes mobility-limited earlier, whereas the anhydride-cured system remains chemically controlled over a broader conversion range. These molecular-level differences directly underpin the distinct curing kinetics observed experimentally and set the basis for the subsequent analysis of the TTT diagrams.

Figure 9 and Table 4 summarize the thermogravimetric (TGA) and derivative thermogravimetric (DTG) results for representative formulations, revealing clear differences in thermal stability associated with the cured network structure. The amine-cured formulation exhibits superior thermal resistance, with higher onset degradation temperatures (*T*_5%_ = 330 °C; *T*_10%_ = 393 °C) and a maximum degradation rate at 485 °C, indicating a more thermally stable network. Although ester groups derived from epoxidized linseed oil are retained in both curing systems, the higher thermal stability of ELO-AM is attributed to the distinct network architecture formed by epoxy–amine curing. This network is predominantly composed of C–N linkages and hydroxyl groups formed through epoxy ring-opening reactions, promoting stronger intermolecular interactions and hydrogen bonding while avoiding the introduction of additional thermally labile ester cross-links. Comparable onset degradation temperatures have been reported for biobased epoxy systems cured with amines.^13^

**Figure 9:**
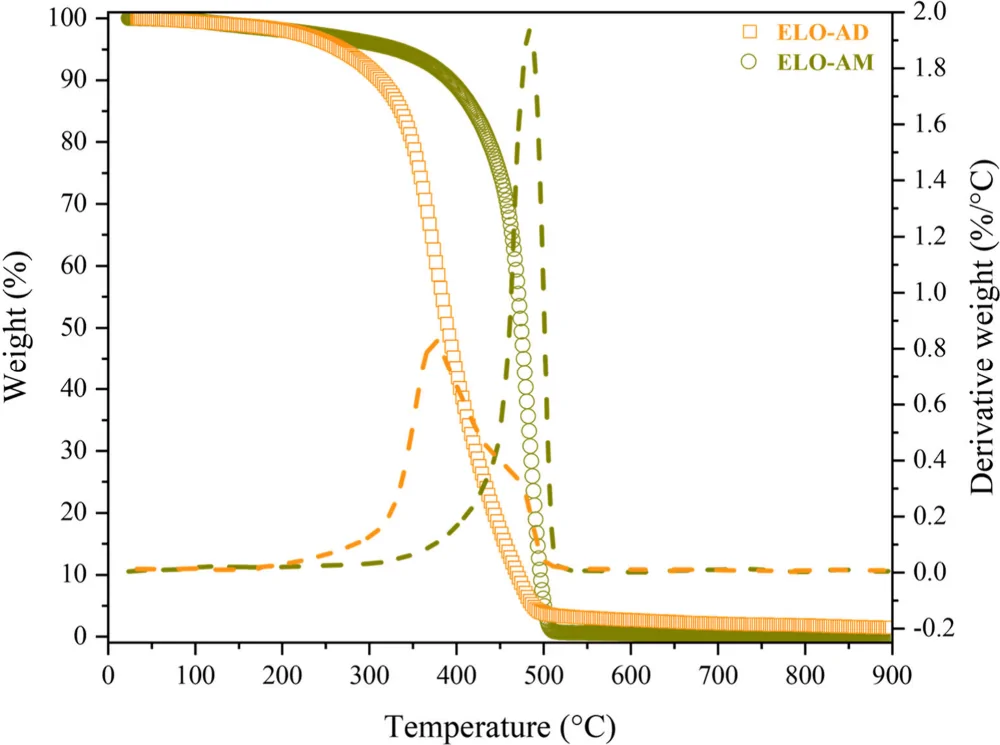
TGA and DTG curves for the ELO-AD and ELO-AM formulations.

**Table 4:** Thermal Degradation Temperatures of the ELO-AD and ELO-AM Formulations

Formulation	*T*_5%_ (°C)	*T*_10%_ (°C)	DTG (°C)	Residual (%)
ELO-AD	266	312	375	1.2
ELO-AM	330	393	485	

The anhydride-cured formulation exhibits lower thermal stability, with *T*_5%_ = 266 °C, *T*_10%_ = 312 °C, and a primary DTG peak at 375 °C, followed by a secondary degradation event around 470 °C. This additional mass-loss stage is consistent with a multistep degradation process involving the cleavage of ester-containing network segments and the subsequent decomposition of more thermally stable structures. The lower thermal resistance of this system agrees with the greater susceptibility of ester linkages to thermal degradation.^9,45^ The ELO-AD formulation also exhibited a small residual mass (∼1.2 wt %) at the end of the TGA experiment. Although the chemical composition of this residue was not directly characterized, its formation is consistent with the presence of aromatic and halogen-containing species in the accelerator, which may promote limited char formation during the final stages of thermal degradation. In contrast, the ELO-AM formulation underwent nearly complete decomposition, leaving no significant residual mass.

### Rheology, Solid Viscoelastic, Mechanical, Swelling, and Gel Content Properties

Figure 10 shows the evolution of viscosity over time for the two curing formulations. In both systems, viscosity increases exponentially as network formation progresses, reflecting the transition from a chemically controlled regime to a mobility-limited state. However, marked differences in the rate and temperature sensitivity of viscosity buildup are observed between the anhydride- and amine-cured systems.

**Figure 10:**
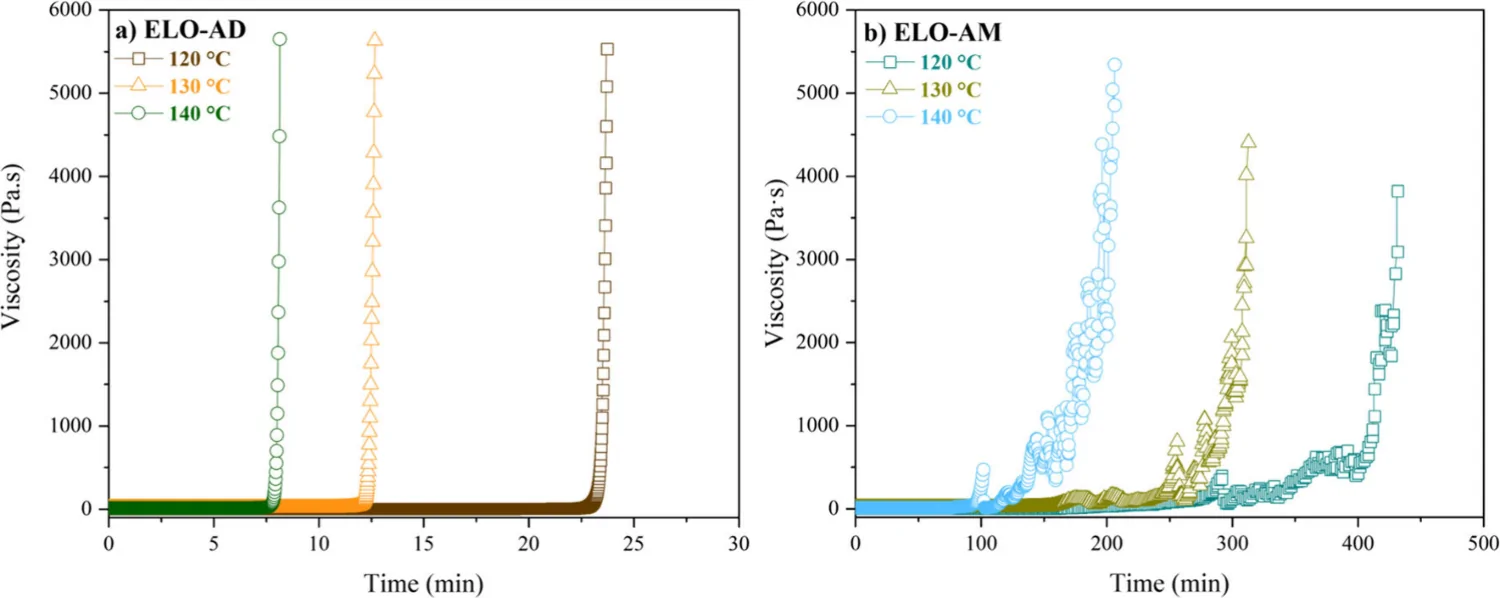
Dynamic viscosity increase at different temperatures for the ELO-AD (a) and ELO-AM (b) formulations.

For the ELO-AD (Figure 10a), increasing temperature leads to a pronounced viscosity rise, indicating rapid network formation once sufficient thermal activation is provided. At 140 °C, the viscosity rises sharply and reaches values of about 6000 Pa·s within 10 min, whereas at lower temperatures the increase is progressively delayed. This strong temperature dependence is consistent with the curing mechanism of epoxy–anhydride systems, in which epoxy ring-opening and esterification reactions are highly temperature-sensitive. In addition, the use of tertiary amines as catalysts promotes rapid development of cross-link density at elevated temperatures, resulting in a steep increase in viscosity associated with fast network consolidation.^46^

The amine-cured formulation (Figure 10b) exhibits a significantly slower rate of viscosity buildup across the entire temperature range. Even at 140 °C, more than 100 min are required to reach viscosities comparable to those attained by the anhydride system within minutes. This behavior cannot be attributed solely to lower intrinsic chemical reactivity; rather, it reflects the combined effects of network architecture and mobility constraints during epoxy–amine curing. Although epoxy–amine reactions proceed readily, competing aminolysis reactions lead to a more flexible network with a lower glass transition temperature, delaying the sharp rise in viscosity. However, as curing advances, diffusion limitations progressively restrict further reaction.

These viscosity profiles corroborate the kinetic and TTT analyses, demonstrating that the anhydride system undergoes rapid, thermally activated network formation leading to early viscosity divergence, whereas the amine system displays a more gradual transition toward the diffusion-limited regime.

The rheological parameters for both formulations are summarized in Table 5. In both systems, the initial viscosity (η_0_) decreases with increasing temperature, while the viscosity growth rate constant (*k*_η_) increases, reflecting enhanced molecular mobility and accelerated network formation at elevated temperatures. The ELO-AD consistently exhibits higher *k*_η_ values than ELO-AM, indicating a faster viscosity buildup and more rapid cure progression. To further quantify the thermal sensitivity of the viscosity evolution, the apparent *E*_a_ associated with *k*_η_ was estimated assuming an Arrhenius-type dependence of the viscosity growth rate constant, *k*_η_=*k*_0_ exp­(−*E*_a_/*RT*), using the experimental data obtained at 120, 130, and 140 °C. Based on the linear fit of ln*k*_η_ vs 1/*T*, the apparent *E*_a_ for viscosity growth was found to be approximately 91 kJ·mol^–1^ for ELO-AD and 62 kJ·mol^–1^ for ELO-AM.

**Table 5:** Rheological Parameters Obtained for the ELO-AD and ELO-AM Formulations

		Temperature (°C)		
Formulation		120	130	140
ELO-AD	η_0_ (Pa·s)	0.015	0.0023	0.0015
	*k*_η_ (min^–1^)	0.2147	0.5012	0.8335
	*R* ^2^	0.97	0.99	0.97
	*t*_gel_ (min)	22.41	11.88	7.56
	α_g_	0.51	0.48	0.40
ELO-AM	η_0_ (Pa·s)	0.0293	0.0084	0.0081
	*k*_η_ (min^–1^)	0.0182	0.0276	0.0454
	*R* ^2^	0.95	0.97	0.99
	*t*_gel_ (min)	406.66	246.67	186.67
	α_g_	0.80	0.85	0.95

The higher apparent *E*_a_ for flow observed for the anhydride-cured system confirms its stronger thermal sensitivity and rapid network consolidation once sufficient thermal activation is provided. In contrast, the lower *E*_a_ associated with the amine-cured system reflects a more gradual viscosity evolution governed by mobility and diffusion constraints, in agreement with its lower *T*_g_ and longer characteristic curing times observed in the TTT diagrams.

The gel time (Table 5) determined from the exponential growth of viscosity enabled the estimation of the apparent *E*_a_ associated with the formation of a continuous three-dimensional network in both systems.^47,48^ Assuming an Arrhenius dependence of the inverse gel time (*k* ∝ 1/*t*_gel_), apparent activation energies of approximately 74 kJ·mol^–1^ (*R* ^2^ > 0.99) for the anhydride-cured system (ELO-AD) and 53 kJ·mol^–1^ (*R* ^2^ > 0.99) for the amine-cured system (ELO-AM) were obtained.

Despite exhibiting a higher *E*_a_, the ELO-AD shows significantly shorter gel times (7–22 min) compared to ELO-AM (186–407 min), demonstrating that the effective kinetic control of gelation is not governed solely by the intrinsic chemical energy barrier, but rather by the interplay between reaction kinetics, segmental mobility, and network structural evolution.

The anhydride-cured system reaches gelation at moderate degrees of conversion (α_g_ ≈ 0.40–0.50), values close to those predicted by percolation theory and Flory–Stockmayer statistics for multifunctional network formation.^48^ Moreover, a slight decrease in α_g_ with increasing temperature is observed, indicating that higher reaction rates promote earlier structural connectivity during network development.

Regarding the anhydride-cured system, showed in Figure 11a, the *T*_g_(*t*) curves display a clear sigmoidal (“S-shaped”) evolution: an initial induction/slow-rise region (low conversion and limited network buildup), followed by a rapid *T*_g_ increase as cross-link density accumulates, and finally a plateau as the network approaches its limiting structure. This profile indicates that over a broad interval (≈20–45 °C and ∼0.05–0.2 months), the kinetic paths remain above *T*_g_ long enough to preserve segmental mobility, allowing the main cross-linking pathway to proceed with minimal transport penalty. Consistently, the transformation (iso-conversion) curves at higher temperatures are steep and converge rapidly toward high conversion once *T*_iso_ > *T*_g_, reflecting reaction-controlled behavior over most of the trajectory. Even as temperature decreases, the *T*_g_ intersection shifts to longer times, but vitrification is delayed relative to the amine case, suggesting that mobility is retained for a longer time into the cure.

**Figure 11:**
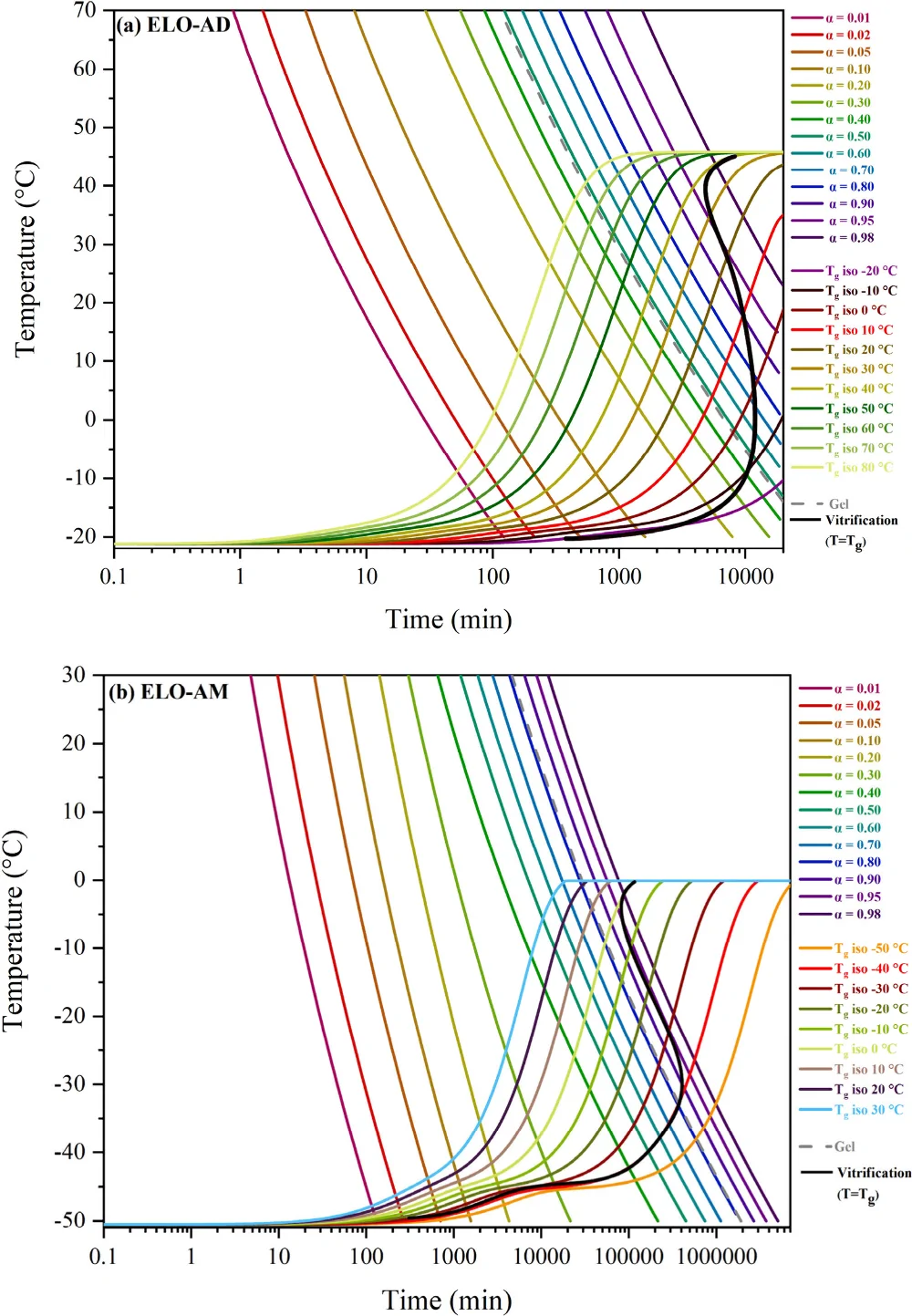
TTT diagram of the (a) ELO-AD and (b) ELO-AM formulations.

In contrast, the amine-cured system, illustrated in Figure 11b, shows a downward-shifted vitrification boundary and *T*_g_ trajectories that intersect transformation curves at lower temperatures (≈−20 to 0 °C) and longer times (∼0.2–1 month). The same S-shaped *T*_g_(*t*) trend is present, but the intersection with *T*_g_ occurs earlier along many isothermal paths, causing an earlier collapse of segmental mobility. This is reflected in the rightward displacement and progressive flattening of the high-conversion transformation curves, which is characteristic of diffusion-limited curing (transport control) at advanced conversion. As *T*_g_ approaches (or exceeds) *T*_iso_, reaction becomes increasingly constrained by reduced free volume and decreased molecular mobility, so additional conversion requires disproportionately longer times.

The relative position of the vitrification line (*T* = *T*_g_) on the transformation curves provides direct visual evidence that ELO-AD remains predominantly temperature- and kinetics-activated, whereas ELO-AM transitions earlier to mobility- and diffusion-governed curing. This interpretation follows the classic Gillham TTT framework, in which gelation and vitrification boundaries partition the cure space into kinetic- and diffusion-controlled regimes, where the “processing window” is determined by how long the system can evolve above *T*_g_ before vitrification arrests the reaction.^49^

Figure 12 presents the solid viscoelastic response of the formulations obtained by DMA as a function of temperature at multiple frequencies. For both systems, increasing frequency shifts the storage modulus (*E*′) curves toward higher temperatures, reflecting the time–temperature equivalence associated with thermally activated segmental dynamics. Accordingly, the glass transition temperatures determined from both *E*′ and tan δ also shift to higher temperatures with increasing frequency, as summarized in Table 6. The ELO-AD exhibits significantly higher stiffness and thermal stability. The *T*_g_ values determined from *E*′ range from 46 to 56 °C, while those obtained from tan δ range from 76 to 94 °C. In addition, *E*′ at 25 °C remains high, ranging from 1167 to 1233 MPa across the frequency range investigated. These results indicate a rigid network with restricted chain mobility, consistent with a higher effective cross-link density promoted by the epoxy–anhydride curing mechanism. The formation of ester-rich linkages and the more compact network architecture contribute to the elevated *T*_g_ and modulus values observed for this system.^8,9^

**Figure 12:**
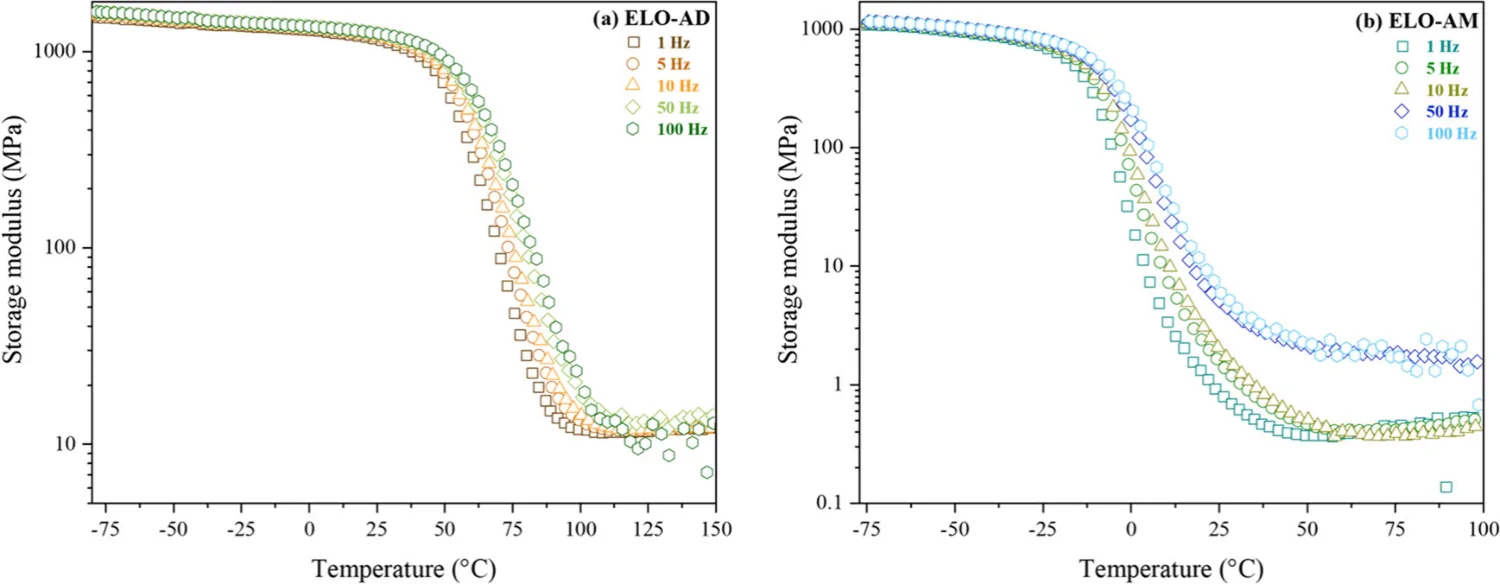
Storage modulus as a function of the temperature at different frequencies for the (a) ELO-AD and (b) ELO-AM formulations.

**Table 6:** Summarized DMA Results Obtained for the ELO-AD and ELO-AM Formulations

		Frequency (Hz)				
Formulation		1	5	10	50	100
ELO-AD	*E*′ (MPa) at 25 °C	1167	1198	1209	1235	1233
	*T*_g_ (°C) from tan δ	76	80	81	88	94
	*T*_g_ (°C) from *E*′	46	49	51	53	56
ELO-AM	*E*′ (MPa) at 25 °C	0.92	1.63	2.04	5.06	5.85
	*T*_g_ (°C) from tan δ	3	8	11	17	19
	*T*_g_ (°C) from *E*′	–16	–15	–12	–11	–10

The ELO-AM displays substantially lower *T*_g_ and modulus values. *T*_g_ determined from E′ ranges from −16 to −10 °C, while *T*_g_ from tan δ spans 3 to 19 °C, indicating a much earlier onset of segmental relaxation. Correspondingly, *E*′ at 25 °C is markedly lower, varying from 0.92 to 5.85 MPa, evidencing a highly compliant material. This behavior is directly related to the chemical structure of the amine curing agent, which contains long aliphatic segments that enhance chain mobility and reduce network rigidity. As a result, the cured material exhibits increased mechanical flexibility but diminished mechanical strength compared to the ELO-AD.^13^ The anhydride-cured system generates a rigid, high-*T*_g_ network that postpones vitrification, enabling the curing process to remain kinetically controlled over a broader conversion range, particularly at elevated temperatures. In contrast, the amine-cured system produces a flexible, low-*T*_g_ network. Despite the higher initial segmental mobility associated with its lower *T*_g_, the evolution of *T*_g_ during curing causes an earlier intersection with the curing temperature, leading to premature vitrification. As a result, the system transitions earlier to a diffusion-limited regime, restricting further conversion at advanced stages.

Regarding the tensile properties, the ELO-AD exhibits the highest stiffness and strength, 2016 ± 226 MPa and 45.8 ± 6.4 MPa, respectively, with a low strain at break of 4.7 ± 1.1%. These results are consistent with the higher *T*_g_, higher glassy storage modulus, and greater cross-link density, confirming the formation of a stiff, highly constrained network. This behavior is associated with the anhydride curing chemistry, which promotes reduced chain mobility and limited deformability.^8^

ELO-AM shows markedly lower mechanical performance, with a modulus of 1.70 ± 0.02 MPa and tensile strength of 0.20 ± 0.01 MPa, but a substantially higher strain at break (∼26.1 ± 3.8%). This reflects the lower *T*_g_, reduced storage modulus, and lower cross-link density of the amine-cured network, resulting from a more open and flexible molecular architecture imparted by the aliphatic amine curing agent.^13^

Cross-link density and swelling behavior provide a direct structural explanation for the kinetic trends and TTT features observed for the curing systems. The ELO-AD exhibits a low swelling degree and no detectable soluble fraction, consistent with its high cross-link density (υ = 1.19 × 10^3^ mol·m^–3^, determined from the rubbery plateau at 1 Hz). This compact and highly interconnected network restricts chain mobility but delays vitrification to higher temperatures, allowing curing to proceed mostly under chemical control over a broader conversion range. As a result, shorter characteristic times are observed in the TTT diagrams once sufficient thermal activation is provided.

ELO-AM forms a less compact, more heterogeneous network, as evidenced by its lower cross-link density (υ = 4.77 × 10^2^ mol·m^–3^), higher swelling ratio (1.69 ± 0.04), and measurable soluble fraction (11.92 ± 0.50%). The presence of extractable species indicates reduced network connectivity and increased intermolecular spacing, thereby enhancing solvent uptake and chain mobility.^31^ However, this structural openness also promotes earlier vitrification under isothermal conditions, intensifying diffusion limitations at advanced conversion and extending the characteristic curing times in the TTT results. The gel content was consistent with the cross-link density of both systems. ELO-AD exhibited a gel content of 97.95 ± 0.16%, indicating a highly interconnected network, whereas ELO-AM showed a lower value of 79.87 ± 4.45%, corresponding to a larger extractable fraction. This behavior agrees with the lower cross-link density of ELO-AM.

These observations confirm that the curing behavior captured in the kinetic analysis and TTT diagrams is primarily governed by network architecture rather than by the intrinsic reaction rate alone. While the amine-cured system exhibits lower activation energies, its lower *T*_g_ and reduced cross-link density promote an earlier transition to diffusion-controlled curing. This behavior arises not only from the intrinsic network flexibility but also from the coexistence of multiple reaction pathways, including epoxy–amine addition and competitive aminolysis of the triglyceride backbone. These concurrent reactions alter network connectivity and contribute to earlier mobility restrictions. In contrast, the anhydride-cured system maintains sufficient segmental mobility over a broader conversion range, enabling faster, more effective curing at processing-relevant temperatures. Similar swelling behavior, in the absence of a soluble fraction, has been attributed to solvent uptake within nanoscale free-volume regions rather than to network disruption.^7^

## Conclusions

This study provides clear experimental evidence that the curing performance of ELO systems is governed by network structure and diffusion constraints rather than intrinsic chemical reactivity alone. Although the amine-cured system exhibits lower activation energies (19–26 kJ·mol^–1^), its low *T*_g_ (−10 to 19 °C), reduced cross-link density (υ = 4.77 × 10^2^ mol·m^–3^), lower gel content (79.87 ± 4.45%), and higher swelling ratio (1.69) indicate a less interconnected network and greater diffusion limitations at advanced conversion, resulting in extended characteristic times in the TTT diagrams.

In contrast, the anhydride-cured system develops a denser network, evidenced by its higher *T*_g_ (46–94 °C), cross-link density (υ = 1.19 × 10^3^ mol·m^–3^), and gel content (97.95 ± 0.16%), together with rapid viscosity growth (*k*_η_ up to 0.83 min^–1^ at 140 °C), resulting in shorter TTT times at processing-relevant temperatures. These kinetic and structural differences translate directly into superior mechanical performance, with a Young’s modulus of ∼2.0 GPa and tensile strength of ∼46 MPa, compared with ∼1.7 and ∼0.2 MPa for the amine-cured system.

The consistent agreement among kinetic modeling, rheology, DMA, gel content, swelling, TTT analysis, and mechanical testing demonstrates that network development and mobility constraints are key determinants of effective curing in biobased epoxy systems. These findings provide a robust framework for selecting curing agents and processing windows to optimize the performance of sustainable thermoset materials.

## Supplementary Material



## Notes

Detailed results of the isothermal DSC analyses for both curing systems; Tables S1 and S2 compiling total enthalpy (Δ*H*_tot_), residual enthalpy (Δ*H*_res_), degree of conversion (α), and glass transition temperature (*T*_g_) for different curing times and temperatures for the ELO-AD and ELO-AM formulations, respectively; complementary data detailing the curing progression under isothermal conditions and supporting the kinetic analysis and TTT construction discussed in the text (PDF)
