# Supplementary material for: Vitrification-Controlled Curing of Epoxidized Linseed Oil Resins: Kinetics, TTT Analysis, and Thermomechanical Performance with Anhydride and Amine Curing Agents
Source: ACS Omega. 2026 Sep 21;11(39):59463–79. doi: 10.1021/acsomega.6c05665 (PMC13647884; doi:10.1021/acsomega.6c05665)
Supplement: Supplementary file 1 [file ao6c05665_si_001.pdf]

Supporting Information

Vitrification-Controlled Curing of Epoxidized Linseed Oil Resins: Kinetics, TTT Analysis, and Thermomechanical Performance with Anhydride and Amine Curing Agents

Cristiane da Silva Fonseca†, Maurício Carpe Diem Ferreira Xavier‡, Otávio Bianchi†, Sandro Campos Amico†, Cesar Liberato Petzhold‡*

† Postgraduate Program in Mining, Metallurgical and Materials Engineering (PPGE3M), Federal University of Rio Grande do Sul (UFRGS), Av. Bento Gonçalves 9500, Porto Alegre, 91501-970, Brazil.

‡ Postgraduate Program in Chemistry (PPGQ), Federal University of Rio Grande do Sul (UFRGS), Av. Bento Gonçalves 9500, Porto Alegre, 91501-970, Brazil.

Corresponding Author*: *otavio.bianchi@gmail.com*

Table S1. Isothermal cure parameters obtained by DSC analyses of ELO-AD.

Temperature (°C)	Time (min)	ΔH_{tot} (J g^{-1})	ΔH_{res} (J g^{-1})	Conversion (α)	T_g (°C)
**80**	7.5	237	234	1	-28
	15	252	234	7	-25
	30	237	219	7	-23
	60	212	183	13	-19
	120	214	141	34	-7
**100**	7.5	235	230	2	-21
	15	215	172	20	-19
	30	198	150	24	-11
	60	204	99	51	-2
	120	247	58	76	3
**120**	7.5	184	166	9	-14
	15	186	104	44	1
	30	145	59	59	6
	60	251	15	93	37
	120	353	-[1]	99	50
**140**	7.5	139	83	40	1
	15	183	25	86	23
	30	211	3	98	38
	60	247	0.27	99	58
	120	295	0.30	99	49

[1] No residual exothermic peaks were detected during nonisothermal.

Table S2. Isothermal cure parameters obtained by DSC analyses of ELO-AM.

Temperature (°C)	Time (min)	ΔH_{tot} (J g^{-1})	ΔH_{res} (J g^{-1})	Conversion (α)	T_g (°C)
**90**	7.5	168	155	1	-58
	15	167	144	1	-54
	30	179	147	2	-53
	60	157	151	4	-52
	120	121	110	8	-49
**110**	7.5	146	144	1	-56
	15	113	111	2	-51

	30	122	118	3	-49
	60	123	117	4	-46
	120	77	64	17	-41
**130**	7.5	117	115	1	-51
	15	82	79	3	-49
	30	94	82	12	-46
	60	83	48	42	-39
	120	88	19	78	-33
**150**	7.5	87	85	2	-49
	15	69	64	6	-45
	30	87	50	41	-40
	60	143	31	78	-32
	120	144	1	99	-25
